# Hydrocarbon Ionomeric Binders for Fuel Cells and Electrolyzers

**DOI:** 10.1002/advs.202303914

**Published:** 2023-10-09

**Authors:** Yu Seung Kim

**Affiliations:** ^1^ MPA‐11: Materials Synthesis and Integrated Devices Los Alamos National Laboratory Los Alamos NM 87545 USA

**Keywords:** electrodes, electrolyzers, fuel cells, hydrogen, ionomers, polymer electrolytes

## Abstract

Ionomeric binders in catalyst layers, abbreviated as ionomers, play an essential role in the performance of polymer–electrolyte membrane fuel cells and electrolyzers. Due to environmental issues associated with perfluoroalkyl substances, alternative hydrocarbon ionomers have drawn substantial attention over the past few years. This review surveys literature to discuss ionomer requirements for the electrodes of fuel cells and electrolyzers, highlighting design principles of hydrocarbon ionomers to guide the development of advanced hydrocarbon ionomers.

## Graphical abstract

Ionomers in the catalyst layers are a critical component for fuel cells and electrolyzers. This review provides the current understanding of the performance‐limiting factor of ionomers from literature over the past three decades. The remaining challenges and design aspects of fluorine‐free hydrocarbon‐based ionomers are discussed to develop more advanced fuel cells and electrolyzers.

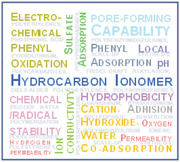

## 1 Introduction

Hydrogen is a light, storable, energy‐dense fuel that produces no direct emissions of pollutants or greenhouse gases. Operating at scale, clean hydrogen from renewable, nuclear, or fossil fuels with carbon capture, utilization, and storage could play a central role in decarbonizing the global energy system.^[^ ^1^ ^]^ Two technologies: electrolyzers and fuel cells, have immediate and vital importance in the hydrogen‐based energy system. Electrolyzers convert electricity into hydrogen, while fuel cells convert hydrogen back to electricity. The performance of both devices primarily depends on electrodes composed of electrocatalysts, catalyst‐supporting materials, and ionomers.

Over the last two decades, extensive efforts have been dedicated to developing advanced electrode materials to improve the performance of fuel cells. Oxygen reduction reaction (ORR) catalysts based on binary alloys of platinum with a transition metal were developed and implemented into commercial fuel cell vehicles.^[^ ^2^ ^]^ High surface area mesoporous carbon‐supporting materials replaced traditional semi‐graphitic carbons for improved performance.^[^ ^3^ ^]^ High oxygen permeable ionomers (HOPI) with a ring structure in the perfluoro polymer backbone enhanced the oxygen transport in the cathode.^[^ ^4^ ^]^ In the next decade, research on electrolyzers built on electrode materials could enable clean hydrogen to be produced at a reduced cost, with a target of $1 per 1 kilogram of hydrogen.

Crucial materials needed for hydrogen‐based energy devices include high‐performing ionomers in the electrodes.^[^ ^5^ ^]^ In the past, ionomer‐focused research was relatively scarce because industrial standard perfluorosulfonic acid (PFSA) ionomers performed well in direct methanol fuel cells (DMFCs) and proton‐exchange membrane fuel cells (PEMFCs). However, as research interest expands to alkaline anion exchange membrane (AEM) fuel cells and electrolyzers, alternative ionomers have drawn substantial attention. Moreover, growing concerns around the regulatory barriers of PFSAs have shifted the direction of research to developing fluorine‐free hydrocarbon ionomers. Several papers, including excellent reviews, have provided information regarding ionomer properties that influence the performance of a specific device.^[^ ^6^ ^]^ However, the peculiar aspects of ionomers were often over‐emphasized or ignored in the papers. This review surveys many papers related to ionomers and examines various aspects of ionomers in fuel cell and electrolyzer applications to provide an insightful overview of hydrocarbon ionomers. This review starts with the history of ionomer development, followed by a discussion of the ionomer's critical properties first from PFSA ionomer studies and then from hydrocarbon ionomer studies. Lastly, the design strategies of hydrocarbon ionomers are discussed.

## 2 History

**Figure 1:**
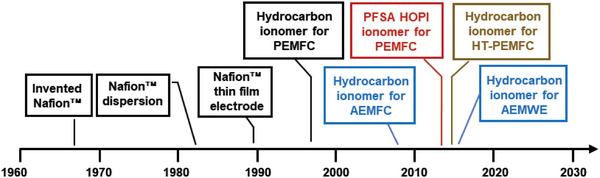
Progression of ionomer development for fuel cells and electrolyzers.

This section briefly summarizes the history of ionomer development for fuel cells and electrolyzers to provide information on how the research focus has changed over time. **Figure** **1** presents a summary of the progression of ionomer development based on the US Department of Energy (DOE), Hydrogen, and Fuel Cell Technologies Office (HFTO) programs.

### 2.1 PEMs and Ionomers for PEMFCs Before 2023

PFSA polymers have been widely used as a membrane for PEMFCs since Nafion was invented in the late 1960s. Before the mid‐2000s, Nafion membranes were produced by extrusion using sulfonyl fluoride precursors which could then be hydrolyzed to the sulfonated polymers. In the mid‐1980s, a procedure for dispersing Nafion in an aqueous media was established by Walther Grot at E. I. Dupont de Nemours and Company.^[^ ^7^ ^]^ This invention led to the use of the PFSA polymer as an ionomeric binder in the catalyst layer in the late 1980s,^[^ ^8^ ^]^ which reduced the platinum catalyst loading of PEMFCs to 0.4 mg cm^−2^ through ionomer thin‐film electrodes. The Nafion dispersion technology also allowed for the processing of dispersion‐cast membranes with more uniform and reduced thickness in the mid‐2000s.^[^ ^9^ ^]^ Short‐side chain PFSA PEMs and dispersions such as Aquivion and 3M ionomers became available in the late 2000s.^[^ ^10^ ^]^

The primary motivation for using hydrocarbon polymers in PEMFC applications before 2003 was the high cost of PFSAs. In the mid‐1990s, styrene‐based PEMs were developed by Ballard Advanced Materials Corporation and Dais Analytic.^[^ ^11^ ^]^ Starting in the late 1990s, sulfonated polyaromatics such as poly(ether ether ketone)s and poly(arylene ether sulfone)s have been investigated.^[^ ^12^ ^]^ Since polyaromatic polymers have good proton conductivity with hydration,^[^ ^13^ ^]^ high radical oxidative stability,^[^ ^14^ ^]^ and low methanol permeability,^[^ ^15^ ^]^ these materials were tested in DMFCs, resulting in higher DMFC performance,^[^ ^16^ ^]^ despite interfacial incompatibility issues between the PEMs and Nafion‐bonded electrodes.^[^ ^17^ ^]^ There were limited studies on the development of hydrocarbon ionomers during this period.

### 2.2 Hydrocarbon PEMs and Ionomers for PEMFCs between 2003 and 2012

In 2003, fuel cell research shifted from DMFCs to hydrogen PEMFCs as the US President's Freedom Cooperative Automotive Research and Fuel Initiatives incentivized the reduction of system complexity, thereby reducing cost and improving reliability. In 2006, Tom Greszler at General Motors reported that automotive fuel cells could be a competitive candidate for internal combustion engines when fuel cell materials enable higher temperature operation, ≈120 °C, 20% RH.^[^ ^18^ ^]^ The US DOE established the High Temperature Membrane Working Group (HTMWG) to develop PEMs with high conductivity at a low RH (0.1 S cm^−1^ at 80 °C, 50% RH).^[^ ^19^ ^]^ The European version of a membrane consortium, i.e., the Coordination Action for Research on Intermediate and high‐temperature Specialized Membrane electrode Assemblies (CARISMA), formed at a similar time (2007).^[^ ^20^ ^]^ After 6 years of research, the HTMWG concluded that the perfluoro imide acids (PFIAs) from the 3M Company have the highest potential to meet the conductivity target at low RH.^[^ ^21^ ^]^ Later, Yandrasits and co‐workers at the 3M Company reported that the PFIA polymers lost bis(sulfonyl)imide at 90 °C at 30% RH, making them unsuitable for prolonged operations under hot and dry conditions.^[^ ^22^ ^]^

No breakthroughs for hydrocarbon PEMs were made during this period, despite some progress being made.^[^ ^23^, ^24^ ^]^ Due to the lack of knowledge on ionomer design, the hydrocarbon PEM materials were used for the ionomeric binders. Effects of the ionomer content, ion exchange capacity (IEC), and microstructure of catalyst layers were the primary topics.^[^ ^6^, ^25^ ^]^ Through the research of this period, the inherently low reactant gas permeability and distinctive features of cyclic voltammograms of hydrocarbon ionomer‐bonded electrodes were recognized. The performance of hydrocarbon ionomer‐bonded electrodes was found to be much inferior to that of the Nafion‐bonded electrodes.

### 2.3 Hydrocarbon Ionomers for AEMFCs between 2008 and 2021

In 2006, the hydrogen PEMFC workshop from national laboratories and universities in the US and Japan reached the consensus that the three most urgent research topics for automotive fuel cells are performance, durability, and cost. One of the approaches to reduce PEMFC cost is to develop anion exchange membrane fuel cells (AEMFCs) where platinum group metal (PGM)‐free catalysts can be used at the cathode without sacrificing substantial performance.^[^ ^26^ ^]^ The US DOE started to support AEMFC research in 2008 and extensive research on hydrocarbon ionomers were done during this period (2008–2016).^[^ ^27^ ^]^ A polyaromatic ionomer developed by Zhuang and co‐workers^[^ ^28^ ^]^ showed superior performance to a perfluorinated anion exchange ionomer^[^ ^29^ ^]^ in AEMFCs, suggesting that hydrocarbon ionomers have potentials for highly performing electrochemical devices.

In 2017, there was a breakthrough in hydrocarbon ionomer development when Mustain and co‐workers prepared radiation‐grafted poly(ethylene‐co‐tetrafluoroethylene) ionomers.^[^ ^30^ ^]^ Although AEMFC performance in their first paper was not impressive (peak power density: 1.2 W cm^−2^ under H_2_/O_2_ conditions), performance rapidly increased within two years to reach a peak power density of 3.4 W cm^−2^ with a poly(norbornene) ionomer.^[^ ^31^ ^]^ Due to the insolubility of the fluoroalkyl and cyclo‐olefinic ionomers, they prepared powder‐form ionomers instead of thin‐film ionomers by grinding in a pestle and mortar and then mixing with catalysts. The high performance with the powder‐form ionomers was puzzling since previous ionomer research for PEMFCs had hypothesized that thin‐ionomer film electrodes prepared from homogeneous ionomer dispersion were advantageous. Another notable approach in 2018–2021 was the use of dissimilar ionomers for the anode and cathode for the imbalanced hydration between the AEMFC electrodes and potential dependent specific ionomer adsorption.^[^ ^32^ ^]^ Substantial knowledge gain achieved through tailoring the chemical structure of hydrocarbon ionomers and interfacial electrochemistry enabled the high performance of AEMFCs.^[^ ^33^ ^]^

### 2.4 PFSA Ionomers for PEMFCs between 2014 and present

While extensive research on the development of PFSA PEMs was performed after 2003 to obtain high proton conductivity at low RH, the importance of ionomers was not well recognized until the mid‐2010s when oxygen mass transport issues with low platinum loading cathode were identified as a key deficiency.^[^ ^34^ ^]^ With this finding, the research for PFSA ionomers shifted to HOPIs in an effort to prepare PFSAs with higher proton conductivity via the introduction of shorter side chains,^[^ ^35^ ^]^ or multi‐acid side chains,^[^ ^36^ ^]^ and more acidic sulfonyl imide groups.^[^ ^37^ ^]^ The most popular approach to preparing HOPIs is the incorporation of bulky moiety in the polymer backbone. The bulky moiety can increase the fractional free volume of polymer to increase oxygen diffusion. Kinoshita and Shimizu from Asahi Glass developed PFSAs with dioxane and dioxolane structures, which increased oxygen transport properties without compromising IEC.^[^ ^38^ ^]^ Others also obtained notably higher performance with PFSA‐based HOPI.^[^ ^39^ ^]^ Another subject of interest in developing advanced PFSA ionomers was reducing specific adsorption of the sulfonate group.^[^ ^40^ ^]^ Lower sulfonated group adsorption by ionic liquids^[^ ^41^ ^]^ motivated the preparation of ionic liquid incorporated hydrocarbon ionomers, i.e., poly(ionic liquid).^[^ ^42^ ^]^

### 2.5 Hydrocarbon Ionomers for AEMWEs between 2016 and Present

The successful launch of hydrocarbon‐based AEMs into AEMFCs motivated the development of high‐performance AEM water electrolyzers (AEMWEs). With glowing interest in green hydrogen production, the US DOE launched the HydroGEN consortium in 2016. The consortium was intended to accelerate the development of materials for AEMWEs, photoelectrochemical hydrogen production, and solar thermochemical hydrogen production. The development of hydrocarbon AEMs and ionomers for AEMWEs started with the materials available for AEMFCs. However, hydrocarbon ionomers from AEMFCs did not always produce high performance in AEMWEs because the operating conditions of AEMWEs, including the level of humidification, operational voltage, and liquid electrolyte circulation, are substantially different. In 2020, Kim and co‐workers showed the high performance of AEMWEs by implementing polystyrene‐based ionomeric binders into the catalyst layers. The current density of PGM‐free anode‐catalyzed AEMWEs reaches 5.3 A cm^−2^ at 1.8 V and 85 °C, which is comparable to the performance of state‐of‐the‐art PEM water electrolyzers.^[^ ^43^ ^]^ Others have also reported high AEMWE performance using hydrocarbon ionomers. Ionomer research for AEMWEs provides valuable information on the electrochemical degradation of ionomers at a high anode potential.

### 2.6 Hydrocarbon Ionomers for HT‐PEMFCs between 2015 and Present

Another critical contribution to understanding hydrocarbon ionomers was achieved with the development of high‐temperature PEMFCs (HT‐PEMFCs). HT‐PEMFCs using conventional phosphoric acid‐doped polybenzimidazole (PBI) PEMs used a polytetrafluoroethylene (PTFE) binder.^[^ ^44^ ^]^ The main reason for using non‐ionomeric binders for HT‐PEMFCs was the extensive flooding due to the redistributed phosphoric acids from the doped PBI membrane.^[^ ^45^ ^]^ However, as ion‐pair coordinated PEMs with a much lower amount of phosphoric acid is developed,^[^ ^46^ ^]^ ionomeric binders play a more pivotal role. Recent studies during 2021–2022 indicated that the rational redesign of phosphonated ionomers improved HT‐PEMFC performance, reaching a peak power density of 0.8 W cm^−2^ at 160 °C under anhydrous H_2_/air conditions.^[^ ^47^ ^]^ The ionomer development for HT‐PEMFCs provides valuable information on mitigating electrode flooding, specific adsorption, and proton conduction. In the next section, the critical properties of ionomers from the PFSA and hydrocarbon ionomer studies are discussed.

## 3 Lessons from the PFSA Ionomer Studies

### 3.1 The Roles of Ionomers in the Catalysts Layers

**Figure 2:**
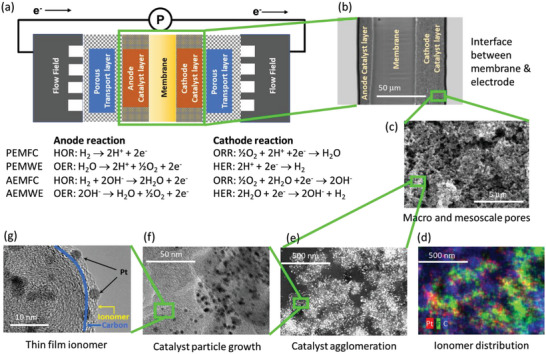
Structure of an MEA (PEMFC), the electrochemical reactions of PEMFC, PEMWE, AEMFC, and AEMWE (a), and micrographic images of cathodes at different length scales (b–g). g) Reproduced with permission.^[^ ^53^ ^]^ Copyright 2012, Elsevier.

**Figure** **2**a shows the schematic diagram of the membrane electrode assembly (MEA) in fuel cells and electrolyzers. The electrochemical reactions of fuel cells and electrolyzers occur in the anode and cathode. On the anode side of the MEA, either hydrogen oxidation reaction (HOR) for fuel cells or oxygen evolution reaction (OER) for electrolyzers occurs. On the cathode side of the MEA, oxygen reduction reaction (ORR) or hydrogen evolution reaction (HER) takes place for fuel cells and electrolyzers, respectively. An ion‐conducting membrane separates the anode and cathode catalyst layers. The outside of the two electrodes is connected to either gas diffusion layers (GDL) for fuel cells or porous transport layers (PTL) for electrolyzers. The transport layers help uniform distributions of the reactants and products for the electrochemical reactions. The transport layers are connected to either the flow field for a single cell or the bipolar plate for a stack. Ionomers are a core component of catalyst layers in MEAs used in fuel cells and electrolyzers. Over the last three decades, several roles of PFSA ionomers in the MEAs were identified in different length scales.

At the micrometer scale, ionomers provide interfacial robustness between the membrane and catalyst layer (Figure 2b).^[^ ^48^ ^]^ Interfacial contact between the membrane and catalyst layer impacts kinetic performance, ohmic resistance, gas and water transport, and crack formation.^[^ ^49^ ^]^ Adhesive properties and dimensional stability under hydrated conditions are two critical properties that prevent interfacial delamination.^[^ ^50^ ^]^ The chemical dissimilarity between the membrane and ionomer is less problematic for strong adhesion, and thus, membrane and ionomer with a dissimilar chemical structure can be used in the same MEA.^[^ ^51^ ^]^

The macroscale (>150 nm) and mesoscale (20–150 nm) pores of electrodes have a substantial influence on the reactant and product transports in MEAs (Figure 2c).^[^ ^52^ ^]^ However, the macroscale or electrode cracks are less desirable for the robustness of electrodes.^[^ ^52e^ ^]^ The morphology of ionomers induced by ionomer‐dispersing agent interactions affects the formation of mesoscale and macroscale pores and cracks.

At the sub‐micrometer scale, early research identified that the agglomerate of carbon‐supported catalysts limits fuel cell performance.^[^ ^54^ ^]^ In fuel cell electrodes, the carbon catalyst supporting the material and the ionomer (20–50 nm) arrange into large aggregates (100–300 nm; Figure 2d).^[^ ^55^ ^]^ Transmission electron microscopy (TEM) and elementary mapping by energy dispersive x‐ray (EDAX) images show that the ionomer distribution within the catalyst agglomerates is highly non‐uniform and locally distributed (Figure 2e).^[^ ^56^ ^]^ The interactions of the catalyst‐ionomer‐dispersing agent in the catalyst ink affect the morphology of the catalyst‐ionomer agglomerate.^[^ ^57^ ^]^ Although the agglomerate morphology impacts water management of the electrode,^[^ ^58^ ^]^ the influence that the uniformity of ionomer distribution on device performance remains largely unknown because of the morphology's complexity and lack of systematic research.

At the 2–10 nm scale, the most pronounced phenomenon is the catalyst nanoparticle growth (Figure 2f).^[^ ^59^ ^]^ Catalyst nanoparticle growth is primarily driven by electrode potentials, but local pH at the vicinity of electrocatalysts determines the dissolution rate of catalyst nanoparticles. At a similar length scale, the ionomer thin film plays a key role in proton conduction, gas transport, and ionomer adsorption (Figure 2g).^[^ ^60^ ^]^ The thickness of the ionomer thin film varies from 2 to 12.5 nm,^[^ ^61^ ^]^ but often lacks ionomer‐coated catalysts, and the aggregation of very large ionomers (>50 nm) is commonly observed at the electrodes.^[^ ^62^ ^]^ Ionomer extrusion into the pores of a high surface area carbon may be difficult since the colloidal particle size of the ionomer is large (> 10 nm).^[^ ^63^ ^]^ Because the properties of ionomer thin film may substantially differ from bulk ionomer properties, extensive studies on ionomer thin films have been performed.^[^ ^64^ ^]^

Because of the complex functions of ionomers in the catalyst layers at different length scales, no single outstanding property correlates with the performance and durability of fuel cells and electrolyzers. Therefore, designing advanced ionomers requires considering several aspects of ionomers and balancing ionomer properties, depending on different electrocatalysts and other materials interactions. The next section explains the key ionomer properties from the PFSA ionomer studies.

### 3.2 Six Key Properties Identified from PFSA Studies

#### 3.2.1 Proton Conductivity

The importance of ionomer's proton conductivity on PEMFC performance was recognized in early studies by Wilson and Gottesfeld where they replaced a non‐conducting PTFE binder with a Nafion ionomer.^[^ ^9^, ^65^ ^]^ The half‐cell polarizations of the devices before mass transport regions were determined by activation (Tafel kinetic parameters) and ohmic polarization characteristics,^[^ ^66^ ^]^

(1) $$E=E_{0}-b\log i-\left(R_{\Omega }i+\;R_{E}i\right)$$

where

(2) $$E_{0}=E_{r}+b\log i_{0}$$

In these equations, *i_0_* is the exchange current density, *b* is the Tafel slope, *E_r_* is the reversible potential for the oxygen electrode reaction, and *R* is predominantly the ohmic resistance in the membrane (*R_Ω_*), and the ionic resistance in the electrode (*R_E_*) responsible for the linear variation of the potential versus current density plot.

Under the assumptions of negligible voltage loss at the anode electrode of PEMFCs, *R_E_* is the effective resistance to proton conduction in the cathode electrode, (*R_H+, cathode_*). The effective resistance in the cathode electrode is proportional to the sheet resistance (*R_sheet_*):

(3) $$R_{H+,\;cathode}=\frac{R_{Sheet}}{3+\zeta }$$

where ζ is the correlation factor.

The *R_sheet_* is a function of the thickness of the catalyst layer (*δ_cl_*), ionomer volume fraction (*ε_i_*), tortuosity (τ), and ionomer conductivity (σ_(T, RH)_):

(4) $$R_{sheet}=\;\frac{\delta_{cl}}{\sigma_{\left(T,\;RH\right)\;}\cdot \;\;\varepsilon_{i}/\tau }$$

These equations show that ion conductivity and the morphology of ionomers directly impact the potential drop of fuel cells. The proton conductivity of Nafion thin film is a function of thickness. For Nafion bulk membranes (N117), the proton conductivity decreases as the film thickness decreases.^[^ ^63^, ^67^ ^]^ The lower proton conductivity of the thin film Nafion is probably due to the anisotropic transport nature of the lamella structure that formed in the thin film.^[^ ^63^, ^68^ ^]^ Other reports indicate that the lower proton conductivity could be due to the constrained hydrophilic/hydrophobic phase separation,^[^ ^69^ ^]^ and oriented sulfonic acid groups.^[^ ^70^ ^]^ Although the conductivity of a thin‐film Nafion is notably lower than bulk conductivity, the proton conductivity of the ionomer‐bonded Pt electrode is 1–2 orders of magnitude higher than non‐ionic PTFE‐bonded Pt‐electrodes, depending on the uniformity and continuity of ionomer thin films.^[^ ^71^ ^]^

#### 3.2.2 Adhesion to Catalyst, Catalyst Support, and Membrane

For the design of the catalyst layer of PEMFCs, the Nafion thin film serves as both the pathway for proton transport and a polymer binder that helps to keep the catalyst nanoparticles intact on the surface of carbon‐supporting materials. At the electrodes, an insufficient amount of ionomer results in poor proton conduction to catalyst particles and a low binding capability. Conversely, having too high of an ionomer content results in mass transport issues. As such, the uniform thin film may provide the best adhesion and proton conduction pathway. However, the thickness of the ionomer thin film in the catalyst layers is mostly highly non‐uniform.

The adhesion energy of the ionomer depends on the oxidation state of the catalyst and carbon‐supporting materials.^[^ ^72^ ^]^ A slightly oxidized surface of platinum and carbon, ≈10%, enhances the binding energy between the hydrated thin film and the substrate. The binding energy of the hydroxylated surface slightly increases with further oxidation but, the binding energy of the epoxidized catalyst and graphite carbon surface decreases, resulting in film delamination. The adhesion energy of the ionomer is closely related to the hydration level. The adhesion between Nafion and the carbon surface decreases with hydration, but the adhesion between Nafion and the Pt nanoparticle increases with hydration. Therefore, with high hydration, the Nafion ionomer becomes more flexible and rearranges on the graphite surface with partial delamination, which allows polymer chains to form bridges between the carbon graphite surface and Pt nanoparticles.

The adhesion energy of the ionomer is also critical for a robust interface between the PEM and catalyst layers.^[^ ^73^ ^]^ Interlaminar delamination between the PEM and catalyst layers was often observed under freeze‐thaw cycling,^[^ ^74^ ^]^ liquid‐fuel feed fuel cells,^[^ ^16^, ^17^, ^75^ ^]^ or the long‐term operation of PEMFCs^[^ ^76^ ^]^ where adhesion of the ionomer from conventional MEA fabrication was insufficient. The interfacial delamination between the PEM and catalyst layers would lead to water accumulation at the interface and high interfacial ion transfer resistance.^[^ ^77^ ^]^ Several stress factors include the uneven compression of the bipolar plate, a non‐uniform electro‐osmotic drag coefficient, and a dimensional mismatch between the water‐swollen PEM and catalyst layer. The difference in volumetric water uptake is a fair predictor, suggesting a corresponding modification of electrodes by varying the ratio of the ionomer binder to the catalyst (I/C ratio) or the type of electrode binder used in the electrode.^[^ ^50^ ^]^

#### 3.2.3 Resistance to Radical‐Induced Oxidative Degradation

The PFSA degradation by reactive free radicals from hydrogen peroxide at the catalyst layer is known to be one of the primary causes that limit PEMFC durability. Small amounts of reactant gases crossing through the membrane generate hydroxyl radicals which attach to the main and side chains of PFSAs during PEMFC operation.^[^ ^78^ ^]^ The prominent sites for attacks in PFSAs include terminal carboxylic acid groups, ether groups in the side chains, tertiary carbon atoms, and the carbon‐sulfur bond of sulfonic acid groups.^[^ ^79^ ^]^ Because radical attacks cause the polymer chain to fragment, mechanical properties, and IECs of the PFSAs reduce. Consequently, the ion conductivity and adhesion properties of the ionomer decrease over time. Therefore, the resistance to radical‐induced degradation is critical for fuel cell durability. Because radical‐induced degradation is more detrimental to the membrane, causing catastrophic failure due to the reduction of molecular weight and IEC, most studies related to the mitigation of radical‐induced degradation focused on incorporating radical scavenger additives into the membrane.^[^ ^80^ ^]^ Cerium is mostly widely used,^[^ ^81^ ^]^ but other inorganic^[^ ^82^ ^]^ and organic radical scavengers^[^ ^83^ ^]^ have been explored for possible implementation. The oxidative stability of reactive oxygen species for sulfonated hydrocarbon membranes has also been extensively investigated using Fentons’ test.^[^ ^84^ ^]^ However, the effectiveness of cerium radical scavenger in hydrocarbon ionomer requires further study as the degradation pathways in Fenton's test and in‐situ fuel cell test may not be the same. The radical‐induced oxidative degradation may occur in quaternized polymers under high pH conditions. Ramani and co‐workers reported that superoxide anion and hydroxyl free radicals can be formed from carbanion with subsequent reduction.^[^ ^85^ ^]^ A subsequent work observed hydroxyl and hydroperoxyl radicals at the cathode and hydrogen radicals at the anode of AEMFCs.^[^ ^86^ ^]^ These oxidative radicals can attack and degrade electron‐rich hydrocarbon moieties such as methoxy or piperidine groups.^[^ ^87^ ^]^

#### 3.2.4 Oxygen Permeability

In the early study, oxygen permeability is identified as one of the major performance‐limiting factors of PEMFCs.^[^ ^88^ ^]^ The permeability of oxygen through a Nafion thin film increases with the water content because the diffusion coefficient of oxygen increases with the water content, but the solubility of the oxygen decreases only a little.^[^ ^89^ ^]^ The importance of oxygen permeability of ionomers was recognized when a low Pt loading cathode suffered from low oxygen supply.^[^ ^90^ ^]^ The high oxygen permeable ionomers are expected to increase oxygen flux to the surface of electrocatalysts.^[^ ^91^ ^]^ HOPI are typically obtained by incorporating cyclic moieties such as dioxolane into the backbone of PFSAs to increase the fractional free volume of the PFSA.^[^ ^38^, ^39^, ^92^ ^]^ The uniformity of the ionomer thin film is critical to oxygen flux. If the coverage of the ionomer has thickness variations, the thicker regions allow only a fraction of the oxygen for the reaction, resulting in high oxygen mass transport resistance.^[^ ^38^ ^]^

#### 3.2.5 Sulfate Anion Adsorption and Nanoparticle Dissolution

While the PTFE backbone of PFSA ionomers is known to be inert to catalyst activity,^[^ ^93^ ^]^ sulfate anions of the polymer can reduce the ORR activity of PEMFCs. Locating the sulfonic acid group near the catalyst surface is advantageous to ORR as protonic access to the catalyst increases,^[^ ^94^ ^]^ but the adsorption of sulfate anions on the surface of Pt catalysts suppresses ORR kinetics.^[^ ^95^ ^]^ Analyses of low molecular weight model anions by cyclic voltammograms and surface‐enhanced infrared absorption spectroscopy indicated adsorbed sulfonated anions on Pt through an oxygen atom of the terminal sulfonated anions.^[^ ^40b^ ^]^ Significantly suppressed ORR by sulfonated anions with relatively low coverage of the ionomer monolayer was observed. The suppression of ORRs becomes less significant as the side chain length decreases due to the reduced chain flexibility of being oriented to the state where ether groups interact with the Pt surfaces. Water content at the Pt‐ionomer interface promotes the desorption of sulfate anions, leading to increased ORR activity at the Pt‐ionomer interface.^[^ ^96^ ^]^

Interactions of catalysts with sulfonic acid groups can accelerate the aggregation of catalyst nanoparticles. The Nafion thin film from a high dielectric constant dispersing agent such as water fully dissociates the sulfonic acid group.^[^ ^97^ ^]^ The highly dissociated sulfonic acid group tends to dissolve catalyst nanoparticles easily, leading to a greater loss of the electrochemical surface area of electrocatalysts. The effects of catalyst ink solvents and ionomers on a commercial high surface area carbon‐supported PtCo cathode catalyst shows that water‐rich inks cause greater loss of Co and concomitant loss of contractions of the PtCo lattice.^[^ ^98^ ^]^ Considering that a low equivalent 3M ionomer (equivalent weight (EW) = 800 g mol^−1^) causes more loss of Co and lattice contractions than the Nafion (EW = 1000 g mol^−1^), sulfonic acid groups of the ionomers in a high‐water content catalyst ink have more interactions with the catalysts. The exposure of sulfonic groups from the thin film also depends on the environment. In a hydrophilic environment such as a platinum oxide surface, sulfonic acid groups tend to expose to the surface.^[^ ^99^ ^]^ A rejuvenating cathode catalyst was developed by flowing hot and drying nitrogen gas to mitigate catalyst aggregation.^[^ ^100^ ^]^

#### 3.2.6 Pore‐Forming Ability

Pore distribution in the electrodes is arbitrarily divided into two regions with a boundary at ≈20 nm, identified as primary pores with a size less than 20 µm and secondary pores having a size greater than 20 µm. The primary pores are related to mass activity (catalyst utilization), and the secondary pores are related to mass transport. The primary and secondary pores are generated by a space within the catalyst agglomerates and a space among the agglomerates, respectively. The porousness of secondary pores in catalyst layers is primarily determined by the interaction between the ionomer and dispersing agent. Early studies with a small angle neutron scattering (SANS) technique suggested that the colloidal Nafion particles in dispersing agents are rod‐like structures.^[^ ^101^ ^]^ Later studies with SANS, nuclear magnetic resonance (NMR), TEM, light scattering, and molecular dynamics indicated that the colloidal morphology of Nafion can vary from rod‐like to lamella or swollen particles, depending on the dielectric constant of the dispersing agent, substrate, and processing conditions.^[^ ^102^ ^]^ The solidification of the Nafion ionomer from dilute dispersion produces three distinctive gelation behaviors, depending on the dispersing agent.^[^ ^103^ ^]^ Water‐based catalyst inks can generate catalyst layers with macro pores and cracks that increase oxygen gas transport.^[^ ^104^ ^]^ However, the mechanical integrity of the catalyst layer is decreased by its highly porous structure. Pure alcoholic dispersion makes Nafion particle dense film with the sulfonic acid group mostly residing inside the particle. Therefore, the electrodes processed from pure alcohol dispersion or aqueous dispersion with a high boiling point alcohol result in high oxygen transport resistance but are more robust under dynamic fuel cell operating conditions. Nafion dispersion in aprotic solvents such as N‐methyl pyrrolidone (NMP) and dimethyl acetamide (DMAc) creates a homogenous gel structure at relatively low concentrations (<15 wt.%) and creates high porosity with mesoscale pores.^[^ ^52^ ^]^ Because of the complexity of morphology formation in different length scales, most studies on Nafion morphologies in the catalyst layers have been done by changing the solvent composition to determine the optimum performance and durability.^[^ ^39^, ^57^, ^105^ ^]^

## 4 Six Additional Key Properties from Hydrocarbon Ionomer Studies

### 4.1 Hydrophobicity in Fuel Cells

**Figure 3:**
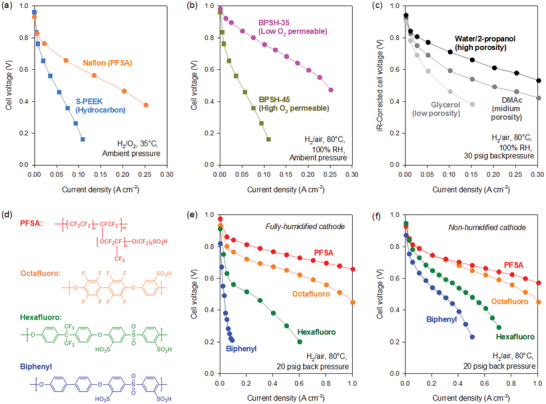
Identification of hydrophobicity as a key ionomer property for LT‐PEMFC performance. a) LT‐PEMFC performance comparison between MEAs using Nafion and a sulfonated poly(ether ether ketone) (S‐PEEK)‐bonded cathode. Reproduced with permission.^[^ ^24a^ ^]^ Copyright 2012, IOP Publishing. b) LT‐PEMFC performance comparison between MEAs using a low O_2_ permeable sulfonated poly(arylene ether sulfone) ionomer (BPSH‐35, P_O2_ = 23 × 10^−12^ mol cm^−2^ s) and high O_2_ permeable ionomer (BPSH‐45, P_O2_ = 32 × 10^−12^ mol cm^−2^ s). c) The effect of a dispersing agent on LT‐PEMFC performance, d) The chemical structure of ionomers with different levels of fluorination, the effect of hydrophobicity of ionomers on PEMFC performance under e) fully humidified cathode and f) non‐humidified cathode. Reproduced with permission.^[^ ^53^ ^]^ Copyright 2012, Elsevier.

Because the fuel cell electrode consists of multiple components, the composition of the electrode is critical for fuel cell performance. Early ionomer studies focused on optimizing the composition of ionomeric binders to produce the best performance.^[^ ^24^, ^106^ ^]^ The optimum weight‐based loading of a hydrocarbon ionomer is 30–60% of a PFSA ionomer primarily due to the lower density (1.0–1.5 g cm^−3^) of hydrocarbon ionomers versus Nafion (density = 2.0 g cm^−3^).^[^ ^107^ ^]^ At optimum loading, the PEMFC performance of hydrocarbon thin‐film electrodes was far inferior to that of PFSA thin‐film electrodes (**Figure** **3**a). Mukerjee and co‐workers performed a thin‐film microelectrode study that suggested that the low oxygen permeability of hydrocarbon thin‐film could limit fuel cell performance.^[^ ^108^ ^]^ The low oxygen permeability of hydrocarbon ionomers was also identified by other researchers.^[^ ^109^ ^]^ Lower hydrophobicity,^[^ ^24^, ^110^ ^]^ and less pore‐forming capabilities^[^ ^111^ ^]^ were also suggested as possible performance limiting factors. Subsequent research indicated that the oxygen permeability of hydrocarbon‐based polyaromatic ionomers is not the primary performance limiting factor as fuel cell performance with ionomers having higher oxygen permeability often showed lower performance (see example in Figure 3b).^[^ ^112^ ^]^ Moreover, no reports show a strong correlation between fuel cell performance and the oxygen permeability of hydrocarbon ionomers.^[^ ^113^ ^]^ As such, increasing the porosity of hydrocarbon ionomers thin‐film electrodes have some effect on fuel cell performance (see example in Figure 3c), but is not a decisive factor. The performance of the PEMFCs with highly porous electrodes was still much inferior to the PFSA‐based PEMFCs.^[^ ^53^ ^]^

On the other hand, the hydrophobicity of hydrocarbon ionomers showed a reasonably high correlation with fuel cell performance.^[^ ^24^, ^114^ ^]^ Kim and co‐workers prepared four cathode electrodes with ionomers having different degrees of fluorination (Figure 3d).^[^ ^53^ ^]^ The degree of hydrophobicity of ionomers decreased in the order of PFSA > Octafluoro > Hexafluoro > Biphenyl. Keeping other MEA components and fabrication conditions the same, the four MEAs with different cathodes were tested under identical operating conditions. Under fully and non‐humidified conditions, the fuel cell performance increased in the order: PFSA > Octafluoro > Hexafluoro > Biphenyl. This result suggests that the hydrophobicity of ionomers may be a major performance‐determining factor (Figure 3e,f). According to Karan and co‐workers, oxygen diffusivity between the gas and the liquid‐water phase is several orders of magnitude different (3 × 10^−5^ m^2^ s^−1^ in the gas phase versus 2 × 10^−9^ m^2^ s^−1^ in the liquid‐water phase),^[^ ^115^ ^]^ suggesting that the high hydrophobicity of electrodes can effectively prevent electrode flooding to improve performance.^[^ ^116^ ^]^

The hydrophobicity of ionomers used for the anode of AEMFCs is more critical than the cathode of PEMFCs because, at a given current density, the amount of water produced at the anode of AEMFCs is two times greater than that at the cathode of PEMFCs. For AEMFCs, water transport by electro‐osmotic drag can additionally increase water content at the anode, particularly, at high current density operation.^[^ ^117^ ^]^ Hence, PEMFCs have an 8–12 H_2_O imbalance per O_2_, but in AEMFCs, the water imbalance is up to 38 water molecules per O_2_, depending on the electro‐osmotic drag coefficient of AEMs.^[^ ^118^ ^]^ Although the AEMFC's cathode is less prone to flooding, a high rate of water back diffusion can cause cathode flooding.^[^ ^119^ ^]^ Thus, using hydrophobic ionomeric binders, but possibly with different degrees of hydrophobicity for both electrodes, may increase performance and durability. Before 2018, only a relatively small number of studies had been performed on this for two reasons.^[^ ^120^ ^]^ Firstly, there was more research on AEMFC cathode development where flooding is minimal. Secondly, the anode of AEMFCs requires high hydrogen flow rate due to cation adsorption, which diminishes anode flooding. After 2018, more hydrophobic ionomers were developed that produced high fuel cell performance. Anode flooding issues were also addressed by fluorine functionalized carbons,^[^ ^121^ ^]^ surface treatment of GDLs,^[^ ^122^ ^]^ and hydrophobic microporous layers.^[^ ^123^ ^]^

**Figure 4:**
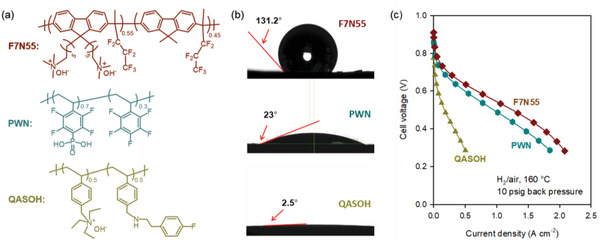
Identification of hydrophobicity as a key ionomer property for HT‐PEMFCs. a) Three ionomers with a different level of fluorination used for the ion‐pair HT‐PEMFCs. b) Water contact angle of the cathode. GDL: W1S1009 (CeTech), Catalyst Pt/C (TEC10E50E, 0.85 mg_Pt_ cm^−2^). c) HT‐PEMFC performance comparison between MEAs having ionomers. Cell compositions: Membrane: Phosphoric acid‐doped hexamethyl trimethyl ammonium functionalized Diels‐Alder polyphenylene (HTMA‐DAPP) (35 µm thickness), electrode ionomer: QASOH, and F7N55, no‐humidification, 148 kPa backpressure. Reproduced with permission.^[^ ^126^ ^]^ Copyright 2022, American Chemical Society.

The lack of hydrophobicity in hydrocarbon ionomers is more of an issue with HT‐PEMFCs where the liquid phase of phosphoric acid in the electrodes substantially reduces oxygen permeability. For conventional HT‐PEMFCs using phosphoric acid‐doped polybenzimidazole, electrodes with hydrophobic non‐ionic binders such as PTFE or polyvinylidene fluoride exhibited the best fuel cell performance.^[^ ^44^, ^126^ ^]^ To improve phosphoric acid retention in the membrane, ion‐pair coordinated membranes composed of phosphoric acid‐doped quaternary ammonium functionalized polymers were developed.^[^ ^46^ ^]^ Due to the high interaction of the ion‐pair (*E*_Int_ = 110 kcal mol^−1^) compared to the acid‐base interaction (18 kcal mol^−1^), the phosphoric acid content in the cathode of the ion‐pair HT‐PEMFCs is substantially lower than that of the phosphoric acid‐polybenzimidazole based HT‐PEMFCs (1.8 mg cm^−2^ for ion‐pair versus 3.2 mg cm^−2^ for acid‐base).^[^ ^125^ ^]^ The low phosphoric acid content in the cathode of ion‐pair HT‐PEMFCs allows the use of ionomers in the catalyst layer, which improves fuel cell performance. However, ion‐pair HT‐PEMFCs still require controlling the hydrophobicity of the ionomer to prevent performance loss driven by cathode flooding. **Figure** **4** compares the ion‐pair HT‐PEMFC performance as a function of the hydrophobicity of ionomers: fluorinated polyfluorene (F7N55), phosphonated poly(fluoro‐phenyl styrene) (PWN), and quaternized polystyrene (QASOH).^[^ ^126^ ^]^ The contact angle of the electrode using the F755, PWN, and QASOH ionomers was 131.2°, 23.0°, and 2.5°, respectively. Note that the fluoroalkyl side chain is much more effective in increasing hydrophobicity than the fluoroaromatic side chain because of high chain mobility. The ion‐pair HT‐PEMFC performance substantially increased when the hydrophilic ionomer (QASOH) was replaced with a hydrophobic ionomer (PWN). By further increasing the hydrophobicity with a more hydrophobic ionomer (F7N55), the performance of HT‐PEMFC improved. However, the degree of improvement is marginal, which is consistent with the fact that optimum hydrophobicity is required to remove the flooding of electrodes.

### 4.2 Alkaline Stability in AEMFC and AEMWE

The chemical stability of quaternized polymers under high pH conditions was recognized as one of the most significant limiting factors for AEMFC durability.^[^ ^127^ ^]^ The alkaline stability of quaternized polymers largely depends on their cationic groups.^[^ ^27^, ^128^ ^]^ Sulfonium and phosphonium groups are known to have limited chemical stability except for phosphoniums with bulky substituted phenyl groups.^[^ ^129^ ^]^ Benzyl trimethyl ammonium (BTMA) is degraded easily due to the hydroxide ion attacks on the α‐carbon at the benzylic position via S_N_2 substitution.^[^ ^128^, ^130^ ^]^ Imidazoliums,^[^ ^131^ ^]^ alkyl ammoniums,^[^ ^132^ ^]^ and piperidiniums^[^ ^33^, ^133^ ^]^ are relatively stable. Both the cationic functional groups and the polymer backbone can be degraded. For example, poly(aryl ether sulfone)s,^[^ ^27^, ^134^ ^]^ poly(phenylene oxide)s,^[^ ^127^, ^135^ ^]^ poly(ether ether ketone)s^[^ ^136^ ^]^ showed rapid degradation rate due to the aryl ether cleavage reactions.^[^ ^27a^ ^]^

**Figure 5:**
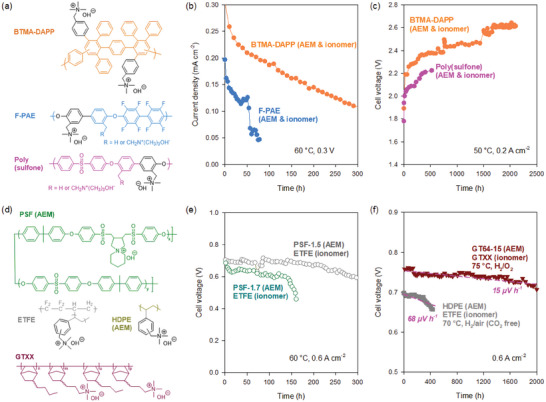
Identification of alkaline stability as a key ionomer property for AEMFCs. a) Chemical structures of polyaromatic ionomers used for AEMFC and AEMWE durability evaluations. b) AEMFC performance comparison between BTMA‐DAPP and F‐PAE‐based cells. Reproduced with permission.^[^ ^27^ ^]^ Copyright 2012, Elsevier. c) AEMWE performance comparison between BTMA‐DAPP and poly(sulfone) based cell. Reproduced with permission.^[^ ^130^ ^]^ Copyright 2022, American Chemical Society. d) Chemical structures of AEMs and ionomers used for AEMFC durability evaluation. e) In situ stability plots showing cell voltage as a function of testing time for MEAs fabricated with PSF‐1.5 and PSF‐1.7 AEM and radiation‐grafted ethylene tetrafluoroethylene (ETFE) ionomer. Reproduced with permission.^[^ ^137^ ^]^ Copyright 2022, IOP Publishing. f) AEMFC durability of an MEA using an HDPE AEM and radiation‐grafted ETFE ionomer and an MEA using a crosslinked norbornene AEM (GT64‐15) AEM and uncrosslinked norbornene ionomer (GTXX, IEC = 1.24 meq g^−1^). For the norbornene‐based MEA, asymmetric ionomers were used for the anode (hydrophilic) and cathode (hydrophobic). Reproduced from CC‐BY open access license.^[^ ^138^ ^]^ Copyright 2019, Royal Society of Chemistry. Reproduced with permission.^[^ ^139^ ^]^ Copyright 2020, John Wiley & Sons.

The degradation behaviors of electrochemical devices are different, depending on the alkaline stability of the AEM and ionomer (**Figure** **5**a). The AEMFC durability of two MEAs using an alkaline stable backbone (BTMA functionalized Diels‐Alder polyphenylene, BTMA‐DAPP) and an alkaline unstable backbone (F‐PAE) was compared (Figure 5b).^[^ ^27^ ^]^ The MEA using a BTMA‐DAPP membrane exhibited >300 h of lifetime. In contrast, the other MEAs using the F‐PAE membrane operated for only ≈70 h due to membrane mechanical failure. Figure 5c compares the durability of two MEAs in DI water‐fed AEMWEs.^[^ ^130^ ^]^ Pivovar and co‐workers also compared the AEMFC performance of two MEAs using spirocyclic polysulfone membranes (PSFs) with two different IECs (Figure 5d).^[^ ^137^ ^]^ The MEA with a lower IEC membrane (PSF‐1.7) showed a slower performance drop, surviving up to 300 h before the cell voltage fell below 0.6 V, compared to the MEA with a higher IEC (PSF‐1.5) (Figure 5e). This result is consistent with others indicating that MEAs using unstable polymer backbones with stable cationic functional groups can operate for several hundreds of hours without catastrophic membrane failures.

Two papers from Mustain and co‐workers provide insights regarding the correlation between the alkaline stability of ionomers and AEMFC performance. In 2019, they maintained 450 h of AEMFC durability at 70 °C under H_2_/CO_2_‐free air conditions using high‐density polyethylene (HDPE)‐AEM and a BTMA functionalized ETFE ionomer (Figure 5f).^[^ ^138^ ^]^ The voltage degradation rate of the AEMFC was 68 µV h^−1^, similar to the PSF cell in Figure 5e using the same ionomer. In 2020, they reported 2000 h of AEMFC durability using an alkaline stable polynorbornene‐based AEM and ionomer (Figure 5f).^[^ ^139^ ^]^ Although the cell operated at a higher temperature (75 °C) and was under H_2_/O_2_ conditions, the polynorbornene‐based MEA showed a significantly lower voltage degradation rate (15 µV h^−1^), suggesting that the alkaline stability of ionomers may affect the voltage degradation rate.

### 4.3 Cation‐Hydroxide‐Water Co‐Adsorption in AEMFC Anode

In 2012, Markovic and co‐workers reported that 3*d*‐transition metal cations and the formation of hydroxides of these elements inhibit the ORR and HOR in alkaline electrolytes through the mass transport limited currents.^[^ ^140^ ^]^ At a similar time, Kohl and co‐workers observed that the adsorption of the tetramethyl ammonium cation on the platinum surface substantially inhibits the oxidation of methanol.^[^ ^141^ ^]^ They also noted that the cation adsorption driven by electrostatic interactions causes cation‐hydroxide accumulation over time. In 2016, McCrum and Janik found that potassium cations can specifically adsorb at all three low‐index facets of the platinum, weakening the binding of hydroxide.^[^ ^142^ ^]^ The potassium‐specific adsorption is more favorable with higher pH, which explains the measured pH dependence shown by the features of cyclic voltammograms for the three low‐index facets of platinum. Subsequent studies on cation adsorption on HOR catalysts were performed to report the effect of catalysts,^[^ ^143^ ^]^ alkali metal cations,^[^ ^144^ ^]^ organic cations,^[^ ^145^ ^]^ and cation functionalized ionomers.^[^ ^146^ ^]^

**Figure 6:**
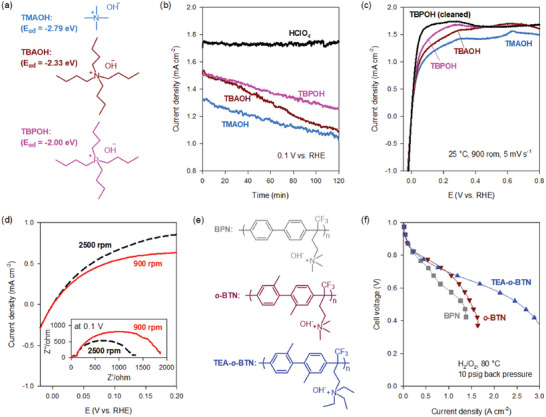
Identification of cation adsorption as a key ionomer property for AEMFCs. a) The chemical structures and adsorption energy of organic cations on Pt(111) surface. b) Chronoamperometry at 0.1 V for the Pt/C in 0.1 m HClO_4_ and alkaline solutions at 25 °C. c) HOR voltammograms of Pt/C in 0.1 m alkaline solutions after the chronoamperometry experiment. Reproduced with permission.^[^ ^147^ ^]^ Copyright 2016, IOP Publishing. d) HOR voltammograms of Pt in 0.1 m TMAOH at two rotating speeds: 900 and 2500 rpm. The inset figure denotes impedance at 0.1 V vs RHE. Reproduced with permission.^[^ ^149^ ^]^ Copyright 2016, American Chemical Society. e) The chemical structure of three ionomers to investigate the effect of H_2_ permeability of the AEMFC anode. f) The impact of the anode ionomer on AEMFC performance. AEM: *m*‐TPN (35 µm thickness); anode: Pt‐Ru/C (0.5 mg_Pt_ cm^−2^); cathode: Pt/C (0.6 mg_Pt_ cm^−2^), 100% RH. Reproduced with permission.^[^ ^153^ ^]^ Copyright 2019, Royal Society of Chemistry.

The adsorption energy of organic cations on platinum catalysts changes depending on their chemical structure (**Figure** **6**a).^[^ ^147^ ^]^ The density functional theory (DFT) study indicates that the adsorption energies of tetramethyl ammonium (TMA), tetra butyl ammonium (TBA), and tetra butyl phosphonium (TBP) cations on Pt(111) surface changes as follows: −2.79 eV (TMA^+^) > −2.33 eV (TBA^+^) > −2.00 eV (TBP^+^). The chronoamperometric experiments at 0.1 V versus RHE [Reverse Hydrogen Electrode] showed that the HOR current density of platinum nanoparticles in TMAOH, TBAOH, and TBPOH decreased over time, which is a remarkable contrast to the stable HOR current density of Pt in 0.1 m HClO_4_ (Figure 6b).^[^ ^147^ ^]^ The HOR voltammograms obtained at the end of the 2 h of the chronoamperometric experiment showed significantly lower HOR activities of Pt in 0.1 m TMAOH (Figure 6c), consistent with the DFT results. Similar HOR activity reduction behavior was observed in the HOR of Pt in KOH solutions, which requires a more complex test protocol for HOR voltammograms in alkaline conditions.^[^ ^148^ ^]^

Electrochemical impedance spectroscopy (EIS) was used to investigate the HOR inhibition mechanism by cation adsorption.^[^ ^149^ ^]^ The EIS data of the Pt rotating disk electrode measured at 0.1 V versus RHE are composed of two semicircles: one larger semicircle at low frequencies (0.1–400 Hz) and one smaller semicircle at high frequencies (400 Hz–100 kHz).^[^ ^150^ ^]^ The semicircle at low frequencies notably decreases with increasing rotating speed (Figure 6d), indicating that the semicircle at low frequencies does not originate from a kinetic‐controlled process but from a diffusion‐controlled process. The high H_2_ diffusion resistance by the cation adsorption is due to the extremely low solubility and diffusivity of H_2_ through the co‐adsorbed layer.^[^ ^151^ ^]^ Neutron reflectometry experiments with Pt film in 0.1 m TMAOH‐water showed that the concentration of TMAOH at the Pt surface at 0.1 V versus RHE is high (TMAOH to water ratio: 5:1) and the adsorbed layer increases with time, supporting HOR inhibition by limited diffusion.^[^ ^152^ ^]^

To prove whether cation adsorption affects hydrogen mass transport, the fuel cell performance of MEAs using three different ionomer structures (Figure 6e) was evaluated.^[^ ^153^ ^]^ The first ionomer was a TMA functionalized poly(biphenyl alkylene) (BPN), which has a low hydrogen diffusion coefficient (4.5 × 10^−9^ m^2^ s^−1^ at 80 °C). The second ionomer was a tetraethyl ammonium functionalized poly(*o*,*o*’‐bitolyl alkylene) (TEA‐*o*‐BTN), which has a dimethyl substitution of the biphenyl group, which increases polymer fractional free volume and has a high hydrogen diffusion coefficient (22.3 × 10^−9^ m^2^ s^−1^ at 80 °C). The third ionomer was tetraethyl ammonium functionalized poly(*o*,*o*’‐bitolyl alkylene) with a high hydrogen permeability (24.2 × 10^−9^ m^2^ s^−1^ at 80 °C) (TEA‐*o*‐BTN). The AEMFC performance of MEAs using the three anode ionomers was compared under H_2_/O_2_ conditions (Figure 6f).^[^ ^153^ ^]^ As expected, the performance of the *o*‐BTN cell is higher than the BPN due to higher gas permeability.^[^ ^154^ ^]^ More importantly, despite similar hydrogen permeability, significant performance occurs with the TEA‐*o*‐BTN ionomer, indicating that using less cation adsorbing ionomers is beneficial for AEMFC performance.

### 4.4 Phenyl Adsorption in an AEMFC anode

**Figure 7:**
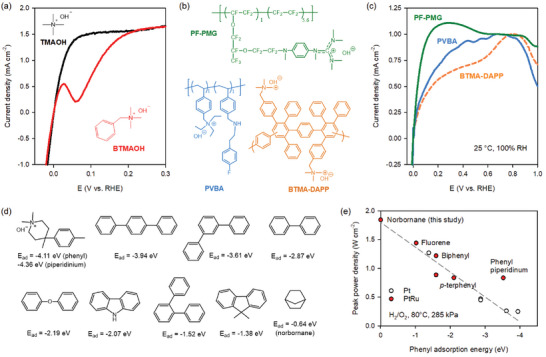
Identification of phenyl adsorption as a key ionomer property for AEMFCs. a) HOR voltammograms of Pt/C in 0.1 m TMAOH and BTMAOH measured at 25 °C; rotating speed: 900 rpm, scan rate: 5 mV s^−1^. Reproduced with permission.^[^ ^156^ ^]^ Copyright 2017, American Chemical Society. b) The chemical structure of ionomers having different amounts of phenyl groups, c) HOR voltammograms of Pt with the three ionomers from microelectrode cells. Reproduced with permission.^[^ ^143^ ^]^ Copyright 2018, American Chemical Society. d) The adsorption energy of phenyl group of different ionomer fragments on a Pt (111) surface. Reproduced with permission.^[^ ^157^ ^]^ Copyright 2017, Elsevier. e) The correlation between phenyl adsorption energy of HOR catalyst and AEMFC performance. All cells used identical (quaternized polyphenylene) AEM and catalysts (anode: Pt/C 0.6 mg_Pt_ cm^−2^ (blank symbol) or PtRu/C 0.5 mg_Pt_ cm^−2^ (red symbol), cathode: Pt/C 0.6 mg_Pt_ cm^−2^) and tests were conducted under the same conditions. Reproduced with permission.^[^ ^159^ ^]^ Copyright 2023, John Wiley & Sons.

The adsorption of phenyl and other aromatic species on electrocatalysts under acidic and alkaline conditions have been known over the past several decades.^[^ ^155^ ^]^ However, the adverse impact of phenyl adsorption on the performance of electrochemical devices was largely ignored until the mid‐2010s, because the predominantly used PFSA ionomers do not have phenyl groups, and other ionomer fragments have little interactions with electrocatalysts. One of the most striking effects associated with ionomer‐catalyst interactions is phenyl adsorption on the HOR catalysts in AEMFCs. **Figure** **7**a shows the effect of phenyl adsorption on the HOR activity of platinum.^[^ ^156^ ^]^ The HOR voltammogram of Pt/C in 0.1 m TMAOH shows a typical HOR shape. On the other hand, the HOR current density of Pt/C in 0.1 m benzyl trimethyl ammonium hydroxide (BTMAOH) decreases at 0.028 V versus RHE because of the phenyl adsorption parallel to the platinum surface. At a higher potential, the HOR current density recovered as the TMA cation adsorption causes the phenyl desorption. The phenyl adsorption from ionomers appears in a wider range of potentials because of the absence of competitive adsorption of the cationic groups. Lower HOR current densities of platinum were obtained with more phenyl group containing ionomers, i.e., HOR current density: perfluorinated phenyl pentamethyl guanidinium (PF‐PMG) > poly(vinylbenzyl ammonium) (PVBA) > BTMAOH functionalized Diels‐Alder polyphenylene (BTMA‐DAPP) (Figure 7b,c).^[^ ^143^ ^]^

The adsorption energies of phenyl piperidinium, *p*‐terphenyl, and phenyl ether are relatively higher than those of carbazole, *o*‐terphenyl, and fluorene (Figure 7d).^[^ ^157^ ^]^ The adsorption energy of phenyl also depends on electrocatalysts. For example, bimetallic PtRu catalysts have a substantially lower adsorption energy than Pt catalysts because of the charge transfer in the *d*‐band center of the bimetallic PtRu.^[^ ^158^ ^]^ The phenyl adsorption on the surface of the HOR catalysts is well‐correlated with the AEMFC performance as shown in Figure 7e.^[^ ^159^ ^]^ One exception is phenyl piperidinium in which high cation adsorption energy inhibits the phenyl co‐adsorption. Although most phenyl adsorption studies have focused on HOR under high pH conditions, phenyl adsorption also negatively impacts HOR under low pH (acidic) conditions. The iR‐corrected current density of the cells using the phenyl‐free Nafion ionomer was 10 mA cm^−2^ at 0.9 V, which is approximately two times higher than the cell using the phenyl‐containing BTMA‐DAPP ionomer.^[^ ^143^ ^]^

### 4.5 Electrochemical Oxidation of Phenyl in an AEMFC Cathode and AEMWE Anode

**Figure 8:**
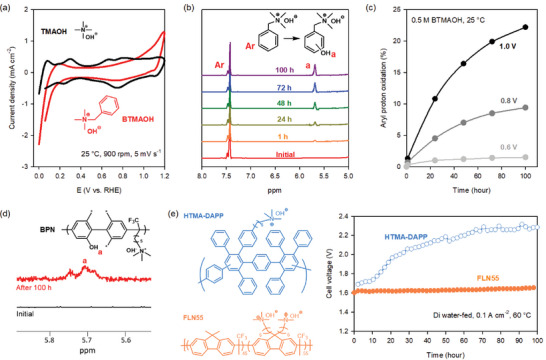
Identification of electrochemical phenyl oxidation as a key ionomer property for AEMFCs and AEMWEs. a) Cyclic voltammograms of Pt/C in 0.1 m TMAOH and BTMAOH. Reproduced with permission.^[^ ^156^ ^]^ Copyright 2017, American Chemical Society. b) ^1^H NMR spectra comparison of the 0.5 m BTMAOH before and after the ORR chronoamperometry at 1.0 V vs. RHE. Reproduced with permission.^[^ ^161^ ^]^ Copyright 2019, Elsevier. c) Aryl proton oxidation conversion of 0.5 m BTMAOH as a function of time and cell potential. d) ^1^H NMR spectra comparison of an anode BPN ionomer before and after the durability test at 2.1 V and 80 °C for 100 h. The * mark denotes other possible oxidation sites. Reproduced with permission.^[^ ^162^ ^]^ Copyright 2017, American Chemical Society. e) The comparison of cell voltage changes of pure water‐fed AEMWEs using HTMA‐DAPP and FLN55 ionomers. Reproduced with permission.^[^ ^165^ ^]^ Copyright 2021, Royal Society of Chemistry.

Another issue associated with the phenyl groups in ionomers is electrochemical oxidation.^[^ ^157^ ^]^ The oxidative current of phenyl groups was observed at potentials above 0.6 V versus RHE in a cyclic voltammogram of Pt/C nanoparticles in 0.1 m BTMAOH (**Figure** **8**a).^[^ ^156^, ^160^ ^]^ To identify the possible degradation product by phenyl oxidation, the structural change of BTMAOH after exposure to 1.0 V versus RHE was investigated by ^1^H NMR. It was found that the phenolic proton peak at 5.68 ppm slowly evolves during the experiment, indicating phenol formation (Figure 8b).^[^ ^161^ ^]^ The electrochemical oxidation rate increases as the cell potential increases (Figure 8c), suggesting that ionomer degradation in the electrolyzer mode would be more substantial than that in fuel cell mode. The phenyl oxidation was also verified with quaternized poly(biphenylene) ionomer (BPN). The ^1^H NMR spectrum of the BPN ionomer obtained from the post‐mortem anode catalyst layer after the electrolyzer test at 2.1 V 80 °C for 100 h shows a phenolic proton peak at 5.75 ppm, indicating phenol formation (Figure 8d).^[^ ^162^ ^]^ Other researchers also observed that the degradation of phenyl‐containing ionomers is much faster at the electrodes exposed to high potentials.^[^ ^163^ ^]^

The ORR and OER activity loss mechanisms were investigated. While the phenyl adsorption at low cell potentials (≈<0.6 V versus RHE) occurs parallel to catalyst surfaces,^[^ ^164^ ^]^ which can block the active sites of catalysts, the phenyl oxidation may occur with different orientations due to the electrochemical potential.^[^ ^156^ ^]^ Therefore, the ORR inhibition by active site blocking may be insubstantial. However, the electrochemical oxidative product, phenol, is detrimental to the alkaline ORR and OER because the phenol group is acidic and changes the local pH in the electrodes. Figure 8e shows the stability of AEMWE using two different ionomers, HTMA‐DAPP and quaternized poly(fluorene) (FLN55). The phenyl adsorption energy of HTMA‐DAPP is higher than fluorene.^[^ ^165^ ^]^ Note that the cell using the HTMA‐DAPP ionomer exhibits a gradual increase in cell voltage over the first 100 h, while the cell using the FLN55 ionomer is stable, confirming that phenyl oxidation plays a critical role in AEMWE durability. Yan and co‐workers confirmed a rapid electrochemical degradation of phenyl. They tested the stability of two model compounds, benzyl‐pyrrolidine and N‐methyl‐1‐heptylpyrrolidine (C_7_Py) cations, in 2 m KOH solution at 80 °C with a constant voltage of 1 V. The benzyl‐pyrrolidine was completely degraded. In contrast, the C_7_Py cation remained stable.^[^ ^166^ ^]^ Recently, Boettcher and co‐workers observed that electrochemical oxidation of ionomers occurred even with phenyl‐free ionomers such as polynorbornene and Nafion under high pH conditions, yet the electrochemical oxidation was greatly suppressed with a liquid alkaline solution, e.g., 0.1 m KOH,^[^ ^167^ ^]^ suggesting that the adsorption of other ionomer fragments and electrochemical oxidation may be the critical performance‐limiting factor for pure‐water‐fed AEMWEs.

### 4.6 Local pH in AEMWEs and CO_2_ Electrolyzers

The concentration of ionic groups in the ionomer determines the local pH at the catalyst layers. The ion dissociation constant (pK) and the concentration of the ionic groups are related to local pH. For the acid system, the acidity of polymer electrolytes is related to proton conductivity.^[^ ^108^, ^168^ ^]^ The impact of ionomer acidity (sulfonic acids) on the performance of electrochemical devices seems to be insignificant. For example, perfluoro imide acids (PFIAs) with higher acid strength than terminal sulfonic acid in the gas phase,^[^ ^169^ ^]^ do not substantially improve device performance.^[^ ^22^, ^170^ ^]^ Likewise, the acidity effect of ionomers on PEMWE is scarce, although pH may affect OER catalyst stability.^[^ ^171^ ^]^

**Figure 9:**
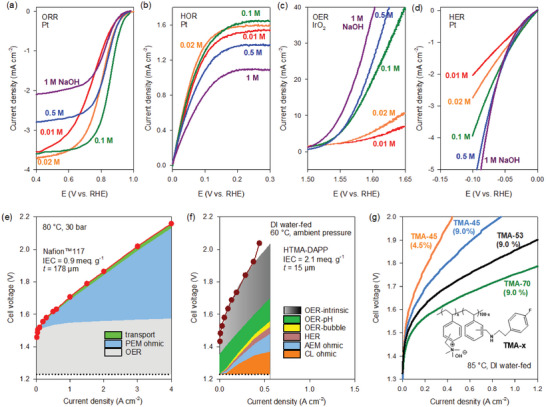
Identification of electrolyte pH as a key ionomer property for AEMWEs. a) ORR, b) HOR, c) OER, and d) HER voltammograms of Pt or IrO_2_ catalysts as a function of NaOH concentration. All curves were recorded with *i*R drop correction at room temperature, 1600 rpm with a scan rate of 10 mV s^−1^. Reproduced with permission.^[^ ^43^ ^]^ Copyright 2020, Springer Nature. e) The voltage breakdown of a PEMWE single cell. Reproduced with permission.^[^ ^173^ ^]^ Copyright 2020, IOP Publishing. f) The voltage breakdown of the DI water‐fed AEMWE single cell. Reproduced with permission.^[^ ^174^ ^]^ Copyright 2021, IOP Publishing. g) The impact of ionomer IEC on AEMWE performance. AEM: HTMA‐DAPP (26 µm thick), ionomer: TMA, anode: IrO_2_ (2.5 mg cm^−2^), cathode: PtRu/C (50 wt% Pt, 25 wt% Ru, 2 mg_Pt_ cm^−2^). Reproduced with permission.^[^ ^43^ ^]^ Copyright 2020, Springer Nature.

For alkaline systems, the local pH of the electrodes impacts AEMWE more than AEMFC.^[^ ^43^ ^]^ Maximum ORR and HOR activity of catalysts for AEMFCs occurs at the intermediate NaOH concentration, ≈0.1 m NaOH (**Figure** **9**a,b). The lower ORR and HOR activity of Pt with the concentrated NaOH solution is explained by cumulative cation‐hydroxide‐water co‐adsorption, which limits the hydrogen access to the catalyst surface.^[^ ^149^ ^]^ By contrast, the OER and HER activity for the AEMWE gradually increased as the NaOH concentration increased from 0.01 m (pH 12) to 1 m (pH 14), suggesting that a high cation hydroxide concentration of ionomers is required for AEMWEs (Figure 9c,d).

The voltage breakdown of PEMWE and AEMWE single cells demonstrates the importance of ionomer pH in AEMWE.^[^ ^172^ ^]^ For PEMWE, the overpotential for the OER kinetics is high in addition to the significant ohmic loss from the thick PFSA membrane (Figure 9e).^[^ ^173^ ^]^ On the other hand, the voltage loss breakdown of deionized (DI) water‐fed AEMWE includes high overpotential due to OER‐pH (Figure 9f).^[^ ^174^ ^]^ Since higher local pH is required for AEMWEs and not AEMFCs, the performance improvement of AEMWEs can be achieved by increasing ionomer content. Figure 9g demonstrates that the AEMWE using a TMA functionalized polystyrene ionomer (TMA‐45, IEC = 2.2 meq. g^−1^) is relatively low at the optimum ionomer content for AEMFC (4.5%). Doubling the ionomer content in the catalyst layer can improve performance and using a TMA ionomer with higher IECs (TMA‐53, IEC = 2.6 meq. g^−1^ and TMA‐70, IEC = 3.3 meq. g^−1^) can further improve AEMWE performance. However, high IEC ionomers have low adhesive properties that lower the durability of the device. Post‐crosslinking or other strategies such as introducing hydrophobic moieties can be considered to balance performance and durability.^[^ ^175^ ^]^

Another essential electrochemical device is the CO_2_ electrolyzer. Numerous papers reported that electrolyte pH plays a key role in determining the faradaic efficiency of CO_2_ reduction reaction (CO_2_RR) products.^[^ ^176^ ^]^ Koper and co‐workers suggest that CO_2_RRs by cobalt protoporphyrin immobilized pyrolytic graphite electrode proceeds through stabilizing a radical intermediate, which acts as Brønstead base.^[^ ^176d^ ^]^ This radical intermediate can abstract a proton from water, leading to an overall reactivity of the CO_2_RR whose pH dependence is different from that of the competing hydrogen evolution. The local pH of current CO_2_ electrolyzers is controlled by the concentration and type of liquid electrolytes, but advanced ionomers may contribute to controlling the local pH and improving CO2RR activity.^[^ ^177^ ^]^

## 5 Design Strategy of Hydrocarbon Ionomer‐bonded Electrodes

### 5.1 General Ionomer Design Approach

**Figure 10:**
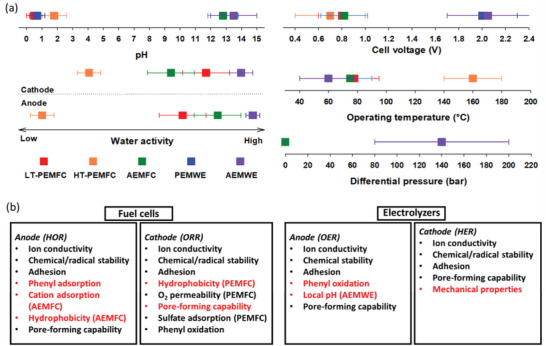
The ionomer property requirement under devices’ operating conditions. a) Operating conditions of fuel cells and water electrolyzers. b) A summary of ionomer requirements for the anode and cathode of fuel cells and electrolyzers. Items in red fonts denote the electrochemical reaction‐specific requirements.

Previous sections discussed twelve critical properties of ionomers that are required for highly performing fuel cells and electrolyzers. Although those properties are generally required for most fuel cell electrodes and electrolyzers, some properties of ionomers are more critical than others depending on specific operating environments. The five most essential working conditions of fuel cells and electrolyzers are i) local pH, ii) cell voltage, iii) water activity, iv) operating temperature, and v) differential pressure. **Figure** **10**a shows the operating conditions of the fuel cells and electrolyzers that need to be considered for ionomer design. PEMFCs and PEMWEs operate at a low pH where oxidative stability of the ionomer is critical. AEMFCs and AEMWEs operate at a high pH where high alkaline stability is required. Particularly, AEMWEs with liquid alkaline supporting electrolytes require greater alkaline stability. The water activity of a PEMFC cathode and AEMFC anode is high so high ionomer hydrophobicity is desirable for fast gas transport. The cell operating voltage of PEMWEs and AEMWEs is high, ≈2 V, so high stability against electrochemical oxidation is required. The anode of PEMWEs and AEMWEs also needs hydrophobicity to remove gas products, although this requirement is not critical as fuel cells. HT‐PEMFCs operate at an elevated temperature, so oxidative degradation of the ionomer may occur relatively fast. If PEMWEs and AEMWEs operate under differential pressure conditions, mechanical properties of the ionomer at the cathode are required.

Based on the property requirement under different operating environments, Figure 10b summarizes the critical properties of ionomers in terms of four electrochemical reactions: HOR, ORR, OER, and HER along with markings for electrochemical reaction‐specific requirements from PFSA and hydrocarbon ionomer research. For fuel cells, ionomers for the AEMFC anode and PEMFC cathode have more required properties. The ionomers for electrolyzers have fewer but more challenging constraints than those for fuel cells such as electrochemical oxidative stability and mechanical robustness.

**Figure 11:**
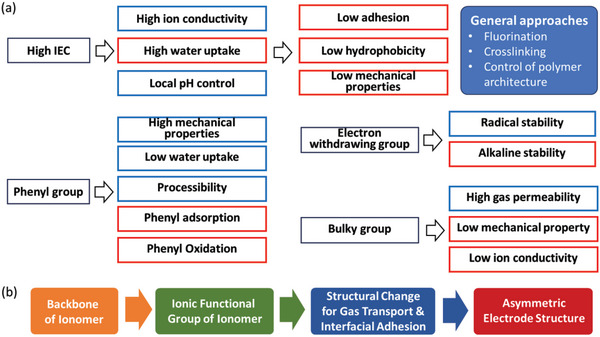
The design strategy of hydrocarbon ionomer‐bonded electrodes. a) Conflicting chemical factors of ionomer. Blue box: positive effect; Red box: negative effect. b) The workflow of the development of high‐performance ionomer‐bonded electrodes.

For high performance, ionomers should meet target performance for every required property. Practically, this is a challenging task because a desired ionomer chemical structure for one property is not good for another property. **Figure** **11**a shows the conflicting chemical structural factors of ionomers. The most known conflicting factor is IEC. Ionomers with higher IEC improve ion conductivity and local pH, but high IEC ionomers result in high water uptake that adversely impact adhesion, hydrophobicity, and mechanical properties. Some approaches such as fluorination, crosslinking, and control of polymer architecture have been attempted to resolve the issue with high water uptake. Other chemical structural factors include incorporating phenyl groups, electron‐withdrawing or donating groups, and bulky groups. Figure 11b shows a workflow of high‐performance ionomer development. The chemical structure of ionomers followed by structural changes to increase transport and interfacial adhesion may be considered. Then, the implementation of ionomer‐bonded electrodes into MEAs can be considered. In the next section, the detailed strategy of high‐performance electrode development is discussed.

### 5.2 Polymer Backbone

#### 5.2.1 Polyaromatics

The first consideration for the design of ionomers is the polymer backbone. The polymer backbone of ionomers is classified as either aliphatic or aromatic. The aromatic backbone provides high ion conductivity, low water uptake, good mechanical properties, high processibility, and a tailored structure.^[^ ^178^ ^]^ However, phenyl groups in the backbone can be oxidized at high electrode potential (>0.6 V vs RHE) or adsorb on the surface of electrocatalysts at low electrode potential (<0.9 V vs RHE), which reduces electrocatalytic activity. Therefore, polyaromatic ionomers have limited use for PEMFCs (cathode),^[^ ^113^, ^179^ ^]^ PEMWEs,^[^ ^180^ ^]^ and AEMWEs.^[^ ^165^, ^181^ ^]^

**Figure 12:**
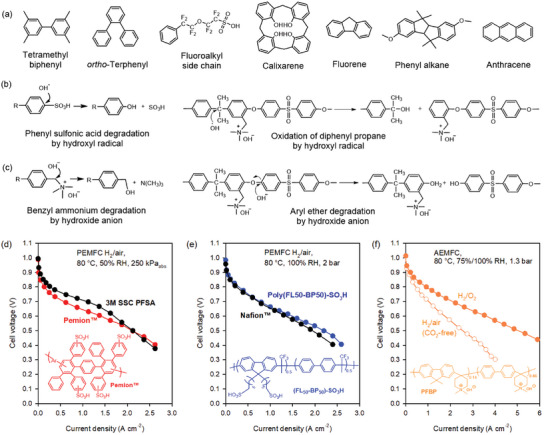
The design strategy of the polyaromatic backbone. a) Bulky functional groups that help reactant gas diffusion. b) Major degradation mechanisms of sulfonated polyaromatics. c) Major degradation mechanisms of phenyl‐based quaternized polyaromatics. d) PEMFC performance of an MEA employing a sulfonated DAPP ionomer (Pemion), PEM: Pemion (7 µm thickness) or 3M short‐sidechain (SSC) PFSA (15 µm thickness), Anode: Pt/C (0.1 mg_Pt_ cm^−2^); cathode: PtCo/C (0.4 mg_Pt_ cm^−2^). Reproduced with permission.^[^ ^114^ ^]^ Copyright 2021, Royal Society of Chemistry. e) PEMFC performance of an MEA employing a superacid catalyzed poly(fluorene) (poly(FL50‐BP50)‐SO_3_H) ionomer. PEM: poly(FL50‐BP50)‐SO_3_H or Nafion 212, Anode: Pt/C (0.2 mg_Pt_ cm^−2^); cathode: Pt/C (0.2 mg_Pt_ cm^−2^). Reproduced with permission.^[^ ^199^ ^]^ Copyright 2023, Royal Society of Chemistry. f) AEMFC performance of an MEA employing a superacid catalyzed poly(fluorene) piperidinium (PFBP) ionomer. AEM: PDTP‐25 (22 µm thickness), Anode: PtRu/C (0.39 mg_Pt_ cm^−2^); cathode: Pt/C (0.26 mg_Pt_ cm^−2^). Reproduced with permission.^[^ ^33^ ^]^ Copyright 2020, John Wiley & Sons.

For polyaromatic ionomers, two design enhancements have been considered. The first is the introduction of functional groups that increase gas permeability. Introducing bulky groups is a common approach.^[^ ^113^, ^179^, ^182^ ^]^ **Figure** **12**a shows examples of bulky groups that have been used to increase oxygen permeability. The second is avoiding aryl ether and phenyl sulfonic acid linkage. According to Ohira and co‐workers, the most energetically favorable (barrierless) reactions of sulfonated polyaromatic materials are i) hydroxyl radical attack of C1 of the C1‐S bond, resulting in the loss of the sulfonic acid, and ii) hydroxyl radical attack of C1 of C1‐O, leading to backbone scission (Figure 12b).^[^ ^183^ ^]^ For the phenyl‐containing ionomers in the cathode of an AEMFC, removing the aryl ether group from the polymer backbone is critical because the aryl ether bond is unstable under high pH conditions (Figure 12c).

The most popular synthetic methods that produce aryl ether‐free polyaromatic ionomers are Diels‐Alder polycondensations and superacid‐catalyzed Friedel Craft reactions. Sulfonated Diels‐Alder poly(phenylene)s (DAPP) were first synthesized by Cornelius and co‐workers in 2005.^[^ ^184^ ^]^ Holdcroft and co‐workers synthesized the sulfonated DAPP using a directly sulfonated monomer (disulfonic acid tetracyclone)^[^ ^185^ ^]^ and started to use it as an ionomer for PEMFCs^[^ ^186^ ^]^ and PEMWEs.^[^ ^180c^ ^]^ Quaternized DAPPs were first synthesized by Cornelius and co‐workers in 2009.^[^ ^187^ ^]^ Later, Hibbs reported a hexamethyl tetramethyl ammonium functionalized DAPP^[^ ^132a^ ^]^ and the polymer was used as an ionomer for AEMFCs^[^ ^143^ ^]^ and AEMWEs.^[^ ^165^ ^]^ The aryl ether‐free poly(phenylene)s by Diels‐Alder polycondensations can produce high‐molecular‐weight fluorine‐free polyaromatics. However, monomers used for directly functionalized reactions are not commercially available and often require multiple synthetic steps. Functionalizing DAPPs by Friedel‐Crafts acylation/benzoylation requires an excess amount of a strong Lewis acid, which can potentially cause side reactions.^[^ ^187^ ^]^ Due to the side reaction with Lewis acids catalysts, the resultant DAPP polymers can be brittle and hard to process. Also, the solubility of the polymer can be low, and thus, only heterogenous amination is possible. Superacid‐catalyzed quaternized polymers were first synthesized using biphenyl, trifluoromethyl alkyl ketones, and trifluoromethanesulfonic acid by Bae and co‐workers in 2015.^[^ ^189^ ^]^ Using the superacid‐catalyzed polycondensation, a variety of polyaromatic ionomers based on terphenyl,^[^ ^132b^ ^]^ fluorenes,^[^ ^190^ ^]^ and phenyl piperidinium^[^ ^133a^ ^]^ were prepared and used for AEMFCs and AEMWEs.^[^ ^143^, ^191^ ^]^ The sulfonated polymers via the acid‐catalyzed polycondensation were reported, but rarely used as an ionomer.^[^ ^168^, ^182^, ^192^ ^]^ Superacid‐catalyzed polycondensation can produce high molecular weight polymers without side reactions. However, tailoring chemical structures is often difficult due to the low reactivity of certain monomers. Also, commercially unavailable monomers need a cost‐intensive purification step. Another significant synthetic route of aryl ether‐free polyaromatic ionomers, although not as popular, is a metal‐catalyzed coupling reaction. Miyatake and co‐workers synthesized several polyaromatic ionomers via coupling reactions and used them as ionomers for PEMFCs^[^ ^23^, ^193^ ^]^ and AEM‐based devices.^[^ ^194^ ^]^ The metal‐catalyzed reactions are versatile techniques but challenges in their practical application include high catalyst cost, difficulty of preparation, sensitivities to air/moisture, and toxicity,^[^ ^195^ ^]^ which may limit their scale‐up applications.

For the anodes of PEMFCs and AEMFCs, biphenyl or terphenyl are not recommended due to strong phenyl adsorption that inhibits HOR efficiency. The phenyl adsorption can be mitigated to a certain degree by using the fluorene group, which has low adsorption energy because of the non‐rotatable phenyl group.^[^ ^185^, ^196^ ^]^ Phenyl piperidinium groups also reduces phenyl adsorption due to stronger adsorption of piperidinium at the HOR potential.^[^ ^157^, ^197^ ^]^

For the AEMWE anode, the use of a polyaromatic ionomer is more challenging due to the electrochemical oxidation of the phenyl group, which reduces the local pH. One possible way to use a polyaromatic ionomer in the AEMWE anode is by introducing liquid electrolytes.^[^ ^198^ ^]^ While polyaromatic backbone ionomers may have electrochemical oxidation at high anode potentials of electrolyzers, several examples show high fuel cell performance and thus a means to replace PFSA‐based ionomers (Figure 12d,e,f).^[^ ^33^, ^114^, ^199^ ^]^ Durability of the fuel cells is the remaining challenge to the widespread use of polyaromatic ionomer technology.

#### 5.2.2 Polyolefins

Polyolefinic backbones resolve electrode issues associated with the phenyl group. Three types of polyolefinic backbone have been considered: aliphatic, fluoroalkyl, and cycloaliphatic. The synthetic process of polyolefinic ionomers is well‐documented.^[^ ^200^ ^]^ Polyolefinic anion exchange polymers were prepared by irradiation‐mediated free radical graft polymerization, metallocene catalyst mediated copolymerization,^[^ ^201^ ^]^ ring‐opening metathesis polymerization (ROMP),^[^ ^202^ ^]^ vinyl‐addition polymerization,^[^ ^203^ ^]^ and Ziegler‐Natta catalyzed copolymerization.^[^ ^204^ ^]^ Polystyrene‐based and radiation‐grafted polyolefinic ionomers have side chain phenyl groups that may have insufficient stability under high pH conditions.

**Figure 13:**
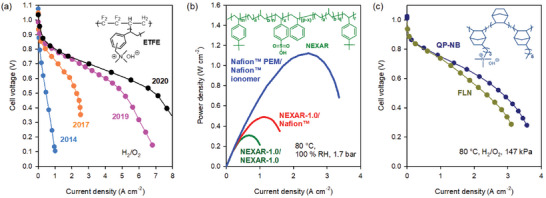
The design strategy of a polyolefinic ionomers. a) AEMFC performance progression of radiation grafted ETFE ionomers. 2014 cell,^[^ ^205^ ^]^ AEM: radiation grafted ETFE (80 µm thickness). Anode: Pt/C (0.4 mg_Pt_ cm^−2^); cathode: Pt/C (0.4 mg_Pt_ cm^−2^); measured at 50 °C, 100 %RH; 2017 cell,^[^ ^30^ ^]^ AEM: radiation‐grafted ETFE (47 µm thickness). Anode: PtRu/C (0.4 mg_Pt_ cm^−2^); cathode: Pt/C (0.4 mg_Pt_ cm^−2^); measured at 60 °C RH = 100 %; 2019 cell,^[^ ^137^ ^]^ AEM: radiation grafted HDPE (21 µm thickness). Anode: PtRu/C (0.4 mg_Pt_ cm^−2^); cathode: Pt/C (0.4 mg_Pt_ cm^−2^); measured at 80 °C RH = 92 %, 2020 cell,^[^ ^211^ ^]^ AEM: poly(norbornene) tetrablock copolymer (10 µm thickness). Anode: PtRu/C (0.7 mg_Pt_ cm^−2^); cathode: Pt/C (0.6 mg_Pt_ cm^−2^); measured at 80 °C, the anode and cathode dew points: 66 and 75 °C, respectively.^[^ ^159^ ^]^ b) H_2_/O_2_ PEMFC performance comparison of MEAs (membrane/ionomer): Nafion NR‐212/Nafion (blue), NEXAR‐1.0/Nafion (red), NEXAR‐1.0/NEXAR‐1.0 (green); measured at 80 °C, 100% RH, backpressure = 1.7 bar. Reproduced with permission.^[^ ^208^ ^]^ Copyright 2021, Elsevier. c) AEMFC performance comparison: AEM: HTMA‐DAPP (25 µm thickness), anode: PtRu/C 0.5 mg_Pt_ cm^−2^, cathode: Pt/C 0.6 mg_Pt_ cm^−2^; measured at 80 °C and 100% RH. Reproduced with permission.^[^ ^159^ ^]^ Copyright 2023, John Willey & Sons.

One outstanding challenge of polyolefins when implementing into the electrodes is their processibility. Compared to polyaromatic ionomers, the solubility of polyolefinic ionomers is low. Several approaches to improve processibility of polyolefinic ionomers have been discussed. The first approach is to make a powder form ionomer to mix with electrocatalysts. In 2014, Varcoe and co‐workers prepared a powder form of a radiation‐graft vinyl benzyl chloride onto ETFE (ETFE‐*g*‐poly(VBTMAC)) ionomer by grinding the ionomer with a pestle and mortar.^[^ ^205^ ^]^ The grinding process made the size of the powder < 100 µm in diameter. The AEMFC performance reported in the paper was low (peak power density = 0.24 W cm^−2^). However, there were remarkable performance improvement (peak power density = 3.5 W cm^−2^) over the next 6 years by operating with a thinner AEM and at a higher operating temperature (**Figure** **13**a). This approach can maximize efficiency of the reactant gas and water permeability at the expense of ionic conductivity and electrochemical surface area. For AEMWE applications, the adhesion of an ionomer powder form to the catalyst nanoparticles may not be enough with more rigorous water activity and high IEC ionomers. To enhance the adhesion, Kohl and co‐workers proposed ionomers with adhesive ester and epoxy components.^[^ ^206^ ^]^

The second approach is to incorporate a polystyrene unit. Holdcroft and co‐workers evaluated various polymer electrolytes containing poly(styrene sulfonic acid) graft chains in 2003.^[^ ^207^ ^]^ In general, graft polymers are hydrophilic, and thus have a very low oxygen permeation rate compared to PFSAs, which renders them unsuitable for most fuel cell applications. Recently, Elabd and co‐workers showed PEMFC performance using pentablock terpolymers with styrene sulfonic acid moiety (NEXAR).^[^ ^208^ ^]^ The best PEMFC performance was obtained with the lowest IEC polymer (IEC = 1.0 meq. g^−1^), yet the performance was still much lower than that of the Nafion‐based cell (Figure 13b) due to the hydrophilic nature of styrene sulfonic acids.^[^ ^209^ ^]^ Balancing the hydrophobicity and solubility of polystyrene is tricky for fuel cell applications. However, one should note that polystyrene‐based ionomers can have high electrolyzer performance that does not need high hydrophobicity in the electrode.^[^ ^43^, ^211^ ^]^

More soluble olefinic ionomers are obtained by increasing the concentration of ionic functional groups.^[^ ^212^ ^]^ Ionic functional groups increase solvent polarity and can disperse polyolefin ionomers. However, homogeneous thin‐film electrodes with high IEC polyolefin ionomers typically have high gas transport resistance making electrode optimization challenging. Introducing bulky side chains into the polycyclic‐olefinic backbone is another way to increase the solubility of the polymers.^[^ ^213^ ^]^ This approach can be effective, but most polyolefin ionomers from this approach have high water uptake at the given IECs, so there is limited literature that have reported high PEMFC performance.^[^ ^201^, ^214^ ^]^ Cyclic olefin copolymers (COCs) are an excellent approach for high hydrophobicity. COCs are low‐density, high hydrophobicity, and thermally/electrochemically stable polymers. Saito and co‐workers prepared polynorbornene‐based thin film electrodes by utilizing norbornene and alkyl bromonorbornene to improve the solubility of the ionomer.^[^ ^215^ ^]^ The soluble ionomer (IEC = 2.5 meq. g^−1^) has a balanced hydroxide conductivity (137 mS cm^−1^ at 80 °C), moderate water uptake (95% at 80 °C), enabling high AEMFC (Figure 13c) and AEMWE performance.^[^ ^159^ ^]^ However, the long‐term stability of the COC ionomers needs to be verified.

### 5.3 Ionic Functional Groups

#### 5.3.1 Anionic Functional Groups

While the industrial standard PFSA uses perfluoro‐sulfonic acids, alkyl or phenyl sulfonic acids are more popular for hydrocarbon ionomers, because of simple synthetic process.^[^ ^168^, ^216^ ^]^ The proton conductivity of a perfluoro sulfonic acid functionalized ionomer is higher than alkyl or sulfonic acid functionalized ionomer at a given weight‐based IECs, yet similar at a given volume‐based IECs.^[^ ^168^, ^217^ ^]^ Because even the protons of the less acidic alkyl sulfonic acid are fully dissociated under hydrated conditions, perfluoro, alkyl, and phenyl sulfonic acids have similar conductivity despite the pK_a_ difference (pK_a_ of CF_3_SO_3_H, C_6_H_5_‐SO_3_H, CH_3_SO_3_H are −14, −6.5, and −1.9, respectively).^[^ ^218^ ^]^ Therefore, the benefits of using a fluoro‐sulfonic acid functional group are relatively small.

Another important family of anionic functional groups is the phenyl phosphonic acids. The benefit of using phosphonic acids over sulfonic acids is anhydrous proton conductivity through structural proton diffusion.^[^ ^219^ ^]^ Due to the high interaction of phosphonic acids, highly phosphonated polymers are brittle, which limits the materials for membrane applications.^[^ ^220^ ^]^ The proton conductivity of phosphonic acid functionalized polymers is low under anhydrous conditions and the possible formation of phosphonic acid anhydride^[^ ^221^ ^]^ further reduces the proton conductivity (< 1 mS cm^−1^ at 100–200 °C). The phosphonic acid anhydride formation can be mitigated by introducing an electron‐withdrawing substituent. In 2021, Kim and co‐workers demonstrated high‐performance HT‐PEMFC with a phosphonated poly(pentafluorostyrene) ionomer (PWN).^[^ ^47a^ ^]^ The HT‐PEMFC using the PWN ionomer showed improved performance compared to the fuel cell using quaternary ammonium functionalized polystyrene, because of enhanced proton conductivity by the phosphonic acid group. Improved performance with the PWN‐type ionomers was also reported by other researchers.^[^ ^222^ ^]^ The proton conductivity of PWN can be further improved by proton transfer from the PFSA, i.e., protonated phosphonic acids.^[^ ^46^, ^47^, ^223^ ^]^ Arges and co‐workers elucidated that the proton conductivity of protonated phosphonic acids is improved due to the suppressed anhydride formation of phosphonic acids.^[^ ^224^ ^]^ Due to the enhanced proton conductivity, the kinetic performance of HT‐PEMFC greatly improved (66 mA cm^−2^ for protonated phosphonic acid ionomer versus 19 mA cm^−2^ for non‐protonated phosphonic acid ionomer at 0.8 V, 160 °C under H_2_/air conditions).^[^ ^47b^ ^]^

#### 5.3.2 Cationic Functional Groups

Alkyl ammonium, phenyl piperidinium, and methyl imidazolium are the most popular cationic functional groups, and several anion exchange ionomers based on these functional groups are commercially available.^[^ ^225^ ^]^ Fuel cell and electrolyzer performance of MEAs employing these ionomers are well documented,^[^ ^33^, ^143^, ^159^, ^181^, ^196^, ^198^, ^226^ ^]^ although performance comparisons of these cationic functional groups are scarce because optimum operating conditions of the MEAs using these ionomers are not identical.

The alkaline stability of cationic groups is probably the most critical consideration for ionomer design.^[^ ^227^ ^]^ The degradation mechanisms of alkyl ammonium cations under high pH conditions are Hofmann elimination and methyl substitution.^[^ ^132c^ ^]^ The alkyl ammonium degradation via Hofmann elimination mainly occurs at low temperatures (≤80 °C) and hydroxide concentration (<4 m NaOH). The alkyl ammonium degradation via methyl substitution becomes the main route of degradation under high temperatures and high hydroxide concentrations. Also, unreacted alkyl bromides may proceed to the cross‐linking reaction by the Williamson ether synthesis route. The main degradation mechanism of phenyl piperidinium is Hofmann elimination.^[^ ^133a^ ^]^ The piperidinium cation attached to the polymer backbone increases the chain strain to speed up degradation.^[^ ^228^ ^]^ A nucleophilic α‐methyl substitution reaction was also identified as a possible degradation route, particularly at a higher KOH concentration.^[^ ^191^, ^229^ ^]^ The chemical stability of imidazolium can be improved by introducing a bulky substituent group at the C2^[^ ^230^ ^]^ and C4,5^[^ ^131^, ^231^ ^]^ positions. The major degradation mechanisms of the bulky imidazolium are the dealkylation of the imidazole ring^[^ ^232^ ^]^ and ring‐opening/C2‐hydroxide attack.^[^ ^233^ ^]^ Coates and co‐workers proposed highly alkaline stable tetrakisaminophosphonium cations stabilized by both resonance and steric hindrance for electrochemical devices that require high alkaline stability.^[^ ^234^ ^]^

The degradation rate of the cationic functional group may increase with low RH, which makes it challenging to operate AEMFCs under drier conditions.^[^ ^235^ ^]^ Dekel and co‐workers compared the alkaline stability of trimethyl benzyl ammonium (TMBA), 6‐azonia‐spiro [5,5] undecane (ASU), and diphenyl imidazolium (BPhIm) at the hydration number (λ) of 0 and found that ASU had an approximately 5.4 times greater degradation rate than BPhIm which in turn had a ≈12 times greater degradation than TMBA.^[^ ^236^ ^]^ However, the testing conditions of the degradation study were very rigorous, and the cationic degradation rate can be much slower under practical fuel cell and electrolyzer operating conditions.^[^ ^232^, ^237^ ^]^

Undesirable adsorption of ammonium and piperidinium affects mostly the hydrogen access for HOR of AEMFCs as discussed in Section 4.3. On the other hand, imidazolium adsorption may also be derived by a conjugated imidazole ring.^[^ ^238^ ^]^ Therefore, the imidazolium adsorption parallel to the catalyst surface may also block the active site of the catalyst. The imidazolium adsorption can be mitigated by supplying liquid electrolytes.^[^ ^226d^ ^]^

### 5.4 Strategies for Enhancing Gas Transport

**Figure 14:**
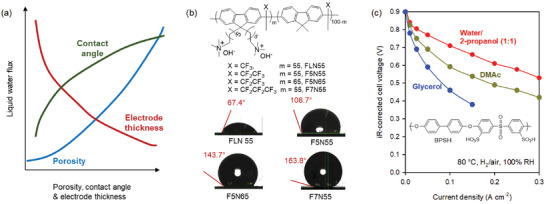
The design strategy of enhancing hydrophobicity of the electrode. a) The effect of electrode thickness, contact angle, and porosity on liquid water flux. b) The effect of ionomer fluorination on water contact angle on the ionomer‐coated gas diffusion layer surface. Catalyst: PtRu/C (0.5 mg_Pt_ cm^−2^, HiSPEC 9100). Reproduced with permission.^[^ ^126^ ^]^ Copyright 2022, American Chemical Society. c) The effect of dispersing agent of catalyst ink on fuel cell performance of MEAs using sulfonated poly(aryl ether sulfone) (BPSH). Reproduced with permission.^[^ ^53^ ^]^ Copyright 2012, Elsevier.

The most common and probably most challenging issue of hydrocarbon ionomer‐bonded electrodes is low gas transport. This issue is more substantial in fuel cells than in electrolyzers. The performance of electrolyzers with a high current density operation is often limited by the insufficient removal of bubbles from electrocatalyst surfaces.^[^ ^174^ ^]^ Gas transport at electrodes is related to the flux of liquid components (water or phosphoric acid) because gas permeability in the liquid phase is much slower than in the gas phase. Liquid component flux is determined by three factors: electrode thickness, hydrophobicity, and porosity. **Figure** **14**a shows the correlation between liquid water flux and the three factors, i.e., electrode thickness, hydrophobicity, and porosity.^[^ ^239^ ^]^ Ionomers substantially affect the hydrophobicity and porosity of the electrodes and marginally affect electrode thickness. This section reviews several strategies to enhance the gas transport properties of hydrocarbon ionomer‐bonded electrodes.

#### 5.4.1 Electrode Thickness Control

For platinum group metal catalyst layers in fuel cells, the optimum electrode thickness is ≈10 µm. Liquid water flux decreases with a thicker electrode which causes a slower reactant gas transport. For low Pt loading catalysts (< 0.2 mg cm^−2^), the thickness of the electrode becomes less than 10 µm, which produces water at high current densities and may cause flooding due to high water concentrations.^[^ ^240^ ^]^ Thus, to maintain the optimum thickness of electrodes, the metal‐to‐carbon ratio needs to be reduced. For Pt‐Co, Pt‐N, or other alloying catalysts, the electrode thickness often exceeds the optimum thickness due to the lower density of the alloying metal component. In this case, a higher metal‐to‐carbon ratio is desirable, although the electrochemically active surface area may decrease due to a larger average particle size.^[^ ^241^ ^]^ For PGM‐free catalyst layers in fuel cells, electrode thickness is much greater (> 50 µm) due to the low volumetric activity of PGM‐free catalysts.^[^ ^242^ ^]^ In such cases, high‐performance fuel cells using hydrocarbon ionomers becomes more challenging.

#### 5.4.2 Hydrophobicity Control

The hydrophobicity of electrodes is typically evaluated by the water contact angle. For LT‐PEMFCs and ion‐pair HT‐PEMFCs, the contact angle of gas diffusion electrodes that prevents water or phosphoric acid flooding is ≈120°.^[^ ^243^ ^]^ For polybenzimidazole‐based HT‐PEMFCs that have 20–30 mg cm^−2^ of phosphoric acid, a much higher hydrophobic electrode (> 150°) is required. Because ionomeric binders are hydrophilic, very hydrophobic non‐ionic binders such as PTFE or PVDF are used for the HT‐PEMFC electrodes.^[^ ^44^, ^244^ ^]^ For DMFCs for which effective CO_2_ removal is required, electrodes with less hydrophobicity (contact angle: 80–120°) showed the highest performance.^[^ ^245^ ^]^

The simplest way to increase the hydrophobicity of electrodes is by decreasing the IEC of the ionomers. Also, decreasing RH helps to remove electrode flooding.^[^ ^32^ ^]^ However, these approaches lower ion conductivity which adversely impacts electrode performance as shown in Figure 11a. Introducing fluorinated groups into the hydrocarbon ionomers is the most common way to decrease RH (Figure 14b).^[^ ^126^ ^]^ PTFE and other fluorinated polymers were often used to increase the hydrophobicity of electrodes.^[^ ^246^ ^]^ Yet, the phase‐segregated fluorine phase may block the active area of catalysts.^[^ ^247^ ^]^ In addition, adding fluorinated moiety into hydrocarbon ionomers still contributes to environmental pollution. Non‐fluorinated groups with high hydrophobicity can be utilized. It is known that alkyl guanidiniums have low hydration energy among other cationic groups.^[^ ^248^ ^]^ Norbornane and other cycloalkanes are known hydrophobic moieties that have been incorporated into the polymer structure.^[^ ^249^ ^]^ Dimethyl siloxane is a common additive to prevent electrode flooding.^[^ ^250^ ^]^ However, one should consider the electrochemical stability of hydrophobic groups before incorporation. For example, alkyl guanidiniums and cyclohexane are less stable.^[^ ^251^ ^]^ Another approach to change the hydrophobicity of ionomers is to control the polymer architecture because the uniformity of ionic group distribution impacts the water uptake of ionomers. Several papers reported that homopolymers or alternating copolymers have notably lower water uptake than random copolymers or block copolymers.^[^ ^252^ ^]^ Also, thin film water uptake can substantially change depending on the substrate.^[^ ^253^ ^]^ This area needs more investigation, but controlling material interactions and ionomer aggregation in the catalyst layer may help increase the hydrophobicity of electrodes.

#### 5.4.3 Porosity Control

Gas diffusion in the electrodes is closely related to the secondary pore structure. The porosity of the electrode is a function of the ionomer loading and dispersing agent.^[^ ^254^ ^]^ Control of secondary pore structures by reducing ionomer loading is limited because minimum ionomer loading is required for ion conduction and ionomer binding capability. Consequently, the most effective way to control porosity is by dispersing agents.^[^ ^255^ ^]^ The impact of dispersing agents on the secondary pore formation has been extensively investigated with PFSAs, as discussed in Section 3.2.6. Hydrocarbon ionomers share features observed in PFSA dispersion, although the solubility (or dispersibility) can be different depending on the chemical structure of the ionomers. Electrodes prepared from hydrocarbon ionomers in water or an aqueous solution with monohydric alcohol have high porosity to produce high fuel cell performance (Figure 14c)^[^ ^53^ ^]^ because the ionic group of the ionomer in water is in the outer particle, which is fully dissociated.^[^ ^97^, ^256^ ^]^ As such, the ionic groups start interacting with each other during the solvent evaporating step, preventing polymer main chain entanglement and producing mechanically fragile but porous structures.^[^ ^103^ ^]^ However, most hydrocarbon ionomers are not dispersible in the aqueous phase. If a hydrocarbon ionomer is dispersible in the aqueous phase, the porosity and mechanical properties of hydrocarbon ionomer‐bonded electrodes need to be controlled by changing the water to mono‐hydrous alcohol ratio and using high boiling point alcohols.^[^ ^105^, ^186^, ^257^ ^]^ Water‐free pure alcoholic dispersing agents can be used to prepare hydrocarbon ionomer dispersion.^[^ ^258^ ^]^ In general, alcoholic dispersing agents with a micelle structure, i.e., a hydrophilic head and a hydrophobic tail, produce a denser structure.^[^ ^103^ ^]^ Therefore, several papers report that electrodes prepared from symmetric alcohols such as isopropanol, di‐propylene glycol, and tetramethyl decyne‐diol showed the highest porosity.^[^ ^259^ ^]^ Aprotic polar solvents such as n‐butyl acetate, DMAc, NMP, and dimethylformamide (DMF) are more effective pore‐forming agents than alcoholic dispersing agents.^[^ ^260^ ^]^ These high boiling solvents may add to monohydric alcoholic dispersion to create a highly porous structure.^[^ ^52b^ ^]^ The capability of secondary pore structures of aprotic solvents was investigated using protonated phosphonic acids.^[^ ^224^ ^]^ It was found that solvents with low pK_a_, such as dimethylsulfoxide (DMSO) (pK_a_ of protonated DMSO = −2.48) push the protons back to the polymer acids, thus keeping them to a more non‐dissociated state, and resulting in less‐porous films. Aprotic solvents with high pK_a_, such as DMAc, DMF, and NMP (pK_a_ of protonated solvents = −0.17 to −0.19) can create porous structures to improve fuel cell performance. Note that the particle growth of electrocatalysts may be inversely proportional to the porosity.^[^ ^261^ ^]^

Another way to control pores in the catalyst layer is to use pore‐forming additives in electrode formulation. In early research (before 2010), removable fillers such as ammonium oxalate and poly(ethylene glycol) were suggested.^[^ ^262^ ^]^ Recently, Wang and co‐workers reported that an ionic covalent organic framework can create mesopores of 2.8 to 4.1 nm that produces 1.6 times higher PEMFC peak power density.^[^ ^263^ ^]^

### 5.5 Strategies for Enhancing Interfacial Adhesion between Membrane and Electrodes

#### 5.5.1 Physical Approaches

To enhance the interfacial contact between membrane and electrodes, several strategies through ionomer modification have been reported. Ionomer modification strategies can be categorized by physical and chemical modification. The most simple and effective physical modification is to increase the MEA processing temperatures.^[^ ^264^ ^]^ This approach works well with ionomers with a T_g_ lower than the processing temperature as the softening of ionomers at the processing temperature can effectively improve the adhesion.^[^ ^265^ ^]^ However, increasing the processing temperature may not be effective for ionomers with a high T_g_. Consequently, alternative methods such as introducing flexible binder material/gradient structure, solvothermal, or wet‐pressing with a small amount of a low boiling point solvent were developed.^[^ ^266^ ^]^ A more sophisticated approach is to form a patterned membrane or electrode that increases the interfacial area, catalyst utilization, lowers ohmic resistance, and improves water management.^[^ ^267^ ^]^ The advantage of this physical approach is the inertness of the electrochemical reaction since little chemical structural changes are accompanied. However, the physical structure at the interface may change over time under the device's operating conditions. Also, the optimal design of the interfacial structure may increase the MEA fabrication cost and process complexity.

#### 5.5.2 Chemical Approaches

For chemical modification, reducing ionomer water uptake is a common method. Several studies on interfacial delamination during freeze‐thaw cycling indicate that ice formation and consequent volume change at the membrane and electrode interface can accelerate the interfacial failure and delamination of the catalyst layer.^[^ ^268^ ^]^ Therefore, decreasing ionomer water uptake by reducing IEC, fluorination, or crosslinking can effectively enhance interface adhesion.^[^ ^197^, ^269^ ^]^ Another popular one is to introduce reactive moieties that can form covalent bonding between membrane and ionomeric binders. Xu and co‐workers reported that polyphenylene ionomers with vinyl benzyl terminal groups formed covalent bonding with the AEM and improved the AEMFC performance and durability by maintaining a tight connection between the AEM and electrodes.^[^ ^270^ ^]^ Yan and co‐workers introduced anthracene‐based in‐situ UV cross‐linkable ionomers to increase the adhesion between catalyst and ionomer.^[^ ^182i^ ^]^

### 5.6 Asymmetric Electrodes

Because the reactants and products of the anode and cathode in a fuel cell or electrolyzer are different, the highest performance of an electrochemical device can be obtained with different electrode compositions and operating conditions. For PEMFCs, the cathode is more prone to flooding because i) the ORR generates water, ii) the electroosmotic drag of water from anode to cathode, and iii) the hydrophilic nature of highly active bimetallic ORR catalysts. For PEMWEs, water is normally supplied to the anode where water is consumed, and thus, water management is relatively easy. Nevertheless, Sung and co‐workers found that the different content of hydrocarbon ionomers in the anode and cathode is beneficial for PEMWE performance.^[^ ^271^ ^]^ Higher ionomer content at the anode (10 wt%) than the ionomer content at the cathode (5 wt%) enhanced the performance by reducing ohmic resistance in the catalyst layer.

**Figure 15:**
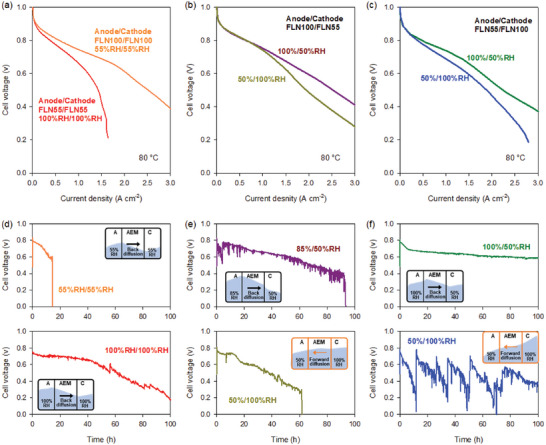
Optimized H_2_/O_2_ AEMFC performance of MEAs using a) symmetric electrode ionomers and b,c) asymmetric ionomers. For symmetric ionomers, either FLN55 or FLN100 ionomers were used for the anode and cathode. For asymmetric ionomers, FLN100 and FLN55 ionomers were used as the anode and cathode, respectively (b) or vice versa (c). 100‐h short‐term test at a constant current density of 0.6 A cm^−2^ of MEAs using d) symmetric electrodes and e) asymmetric FLN100/FLN55 electrodes, and f) asymmetric FLN55/FLN100 electrodes. Performed the 100‐h tests under the humidification that showed the best cell performance. Reproduced with permission.^[^ ^32^ ^]^ Copyright 2012, Royal Society of Chemistry.

For AEMFCs, the water imbalance between the anode and cathode from the reactions is greater, and therefore, water management is more challenging. Using different ionomers at the anode and cathode, i.e., asymmetric electrodes is more effective in AEMFCs in which water management is more challenging. Kim and co‐workers investigated the effect of ionomer IEC on AEMFC performance and durability.^[^ ^32^ ^]^ First, they found that high IEC ionomers require less humidification as high IEC ionomers can hold more water in the electrodes. Under lower humidification, ≈55% RH, the MEA using a high IEC ionomer (FLN100, IEC = 3.5 meq. g^−1^) has higher performance than the MEA using a low IEC ionomer (FLN55, 2.5 meq. g^−1^) under fully hydrated conditions (100% RH) (**Figure** **15**a). The low RH operation with the high IEC ionomer is also beneficial for water management. When asymmetric ionomers were used for the anode and cathode, comparable performance can be obtained by adjusting the anode and cathode humidification. The water activity of the anode can be higher than that of the cathode (back diffusion) or vice versa (forward diffusion). Even though maximum performance from the asymmetric electrodes under the optimized humidification is not much better than the MEA using symmetric electrodes (Figure 15b,c), water management of the MEAs using asymmetric electrodes can be better over time. Asymmetric electrodes with back diffusion showed superior performance stability than symmetric electrodes or asymmetric electrodes with forward diffusion. (Figure 15d,e,f). Mustain and co‐workers also found that the high performance and durability of AEMFCs can be obtained with asymmetric electrodes^[^ ^133^ ^]^ with high IEC ionomers at the anode with lower humidification and low IEC ionomers at the cathode along with higher humidification. The asymmetric electrode configuration was opposite to the MEA prepared by Kim and co‐workers^[^ ^32^ ^]^ but the same principle between humidification level and IEC was applied to manage water transport.

An electrode design with asymmetric ionomers was also reported for AEMWE in which high‐performance PGM‐free catalysts are widely used, and the activity of electrocatalysts is highly dependent on pH (see Section 4.6).^[^ ^272^ ^]^ Several studies show that the ionomer content for the PGM‐free anode catalyst layers needs to be higher for optimized performance and durability,^[^ ^198^, ^273^ ^]^ while the ionomer content of the cathode catalyst is less‐sensitive to the device's performance. However, the choice of high‐performance ionomers for AEMWEs can be flexible when supporting liquid electrolytes are used. The local pH of the electrolyte can be adjusted by the supporting liquid electrolyte, and the adsorption of phenyl or other ionomer fragments has relatively less impact on performance. While a wide range of ionomers, including Nafion or even ionomer‐free electrodes^[^ ^274^ ^]^ can be used, employing well‐designed quaternary ionomers enables further improved performance and durability.

**Figure 16:**
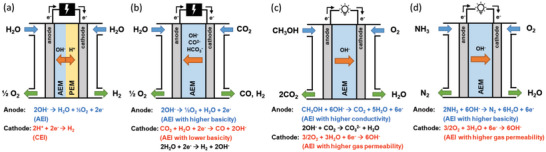
Working principles of asymmetric electrodes in other fuel cells and electrolyzers: a) bipolar membrane water electrolyzer, b) CO_2_ electrolyzer, c) DMFC, d) direct ammonia fuel cell.

For bipolar membrane water electrolyzers^[^ ^226^, ^275^ ^]^ and CO_2_ electrolyzers,^[^ ^276^ ^]^ asymmetric electrodes are a must since OER at the anode requires high pH, but HER for the bipolar membrane water electrolyzers and CO2RR for the CO_2_ electrolyzers require acidic or near neutral environments, respectively. For both water and CO_2_ electrolyzers, supporting electrolytes are normally used to control pH. For direct alcohol/ammonia fuel cells, asymmetric electrodes are optional to enhance ion conductivity and material transport at the anodes and increase oxygen permeability at the cathodes.^[^ ^277^ ^]^ Ionomers with higher IEC can be used at the anodes, and ionomers with higher hydrophobicity can be used at the cathodes. **Figure** **16** summarizes the working principles of asymmetric electrodes in the various fuel cells and electrolyzers.

## 6 Conclusion

Since the first Nafion‐based thin film electrodes were developed for low‐platinum loading PEMFCs in the late 1990s, there has been a continuous interest in developing cost‐effective hydrocarbon ionomers. Recent pressure to avoid perfluoroalkyl substances in electrochemical devices has increased the demand for high‐performance non‐fluorinated ionomers. This review surveys the current understanding of ionomer design in the electrodes of fuel cells and electrolyzers and provides design strategies for hydrocarbon ionomers.

This review echoes previous review articles^[^ ^178^, ^278^ ^]^ that ion conductivity, minimum interaction with electrocatalysts, high adhesion, and high chemical stability under low or high pH conditions are critical properties of ionomers. This review provides additional information in terms of each electrode of fuel cells and electrolyzers. For PEMFCs, ionomer design for high oxygen permeability in the cathode is the key property. For AEMFCs, ionomer design for high hydrogen permeability of the anode needs is the key property. This review shows how researchers approached improving the gas permeability of electrodes with ionomers with high hydrophobicity, secondary pore formation, and minimized cation adsorption for the AEMFC anode. Ionomer design for electrolyzers has fewer problems with reactant water transport. However, this review points out that the electrochemical oxidation of phenyl groups in the anode needs to be dealt with. When it comes to the design of polyaromatic ionomers, a polymer backbone with less phenyl adsorption energy such as polyfluorenes may be used. However, no stable long‐term performance (> 1000 h) of polyaromatic ionomers requires using liquid electrolytes. Polyolefinic ionomers may be a better choice, and more research needs to be done to optimize soluble polyolefinic ionomers. Polyolefinic ionomers are also a good choice for fuel cell anodes where phenyl adsorption can inhibit the HOR activity.

As discussed, ionomers for anode and cathode catalyst layers often require slightly different properties due to different electrochemical reactions. Also, practical systems prefer to humidify one electrode for fuel cells or supply water to one electrode for electrolyzers, which generate water diffusion in MEAs. Therefore, in principle, asymmetric electrodes which use two different ionomers at the anode and cathode, may produce better device performance. This principle will remain important in other electrochemical devices such as CO_2_ electrolyzers and direct alcohol/ammonia fuel cells.

For ionomer studies, more research on structure‐property‐performance is required. The chemical structure of ionomers is of vital importance for the proper functioning of electrochemical reactions. Control of the electrode structure by ionomers is equally important but largely unexplored. The scalability of ionomers may be less critical until the best device performance is realized. Overall, the impact of high‐performance ionomers on the performance and durability of electrochemical devices is high as ionomer development is far behind electrocatalyst development.

## Conflict of Interest

The authors declare no conflict of interest.
